# Genetic Analysis of Hematological Parameters in Incipient Lines of the Collaborative Cross

**DOI:** 10.1534/g3.111.001776

**Published:** 2012-02-01

**Authors:** Samir N. P. Kelada, David L. Aylor, Bailey C. E. Peck, Joseph F. Ryan, Urraca Tavarez, Ryan J. Buus, Darla R. Miller, Elissa J. Chesler, David W. Threadgill, Gary A. Churchill, Fernando Pardo-Manuel de Villena, Francis S. Collins

**Affiliations:** * Genome Technology Branch, National Human Genome Research Institute, National Institutes of Health, Bethesda, Maryland 20892; † Department of Genetics, The University of North Carolina, Chapel Hill, North Carolina 27599; ‡ Oak Ridge National Laboratory, Oak Ridge, Tennessee 37831; § The Jackson Laboratory, Bar Harbor, Maine 04609; ** Department of Genetics, North Carolina State University, Raleigh, North Carolina 27695

**Keywords:** Mouse Genetic Resource, Mouse Collaborative Cross, hematology, hemoglobin β, mean red cell volume, QTL, mouse genetics, complex traits, shared ancestry

## Abstract

Hematological parameters, including red and white blood cell counts and hemoglobin concentration, are widely used clinical indicators of health and disease. These traits are tightly regulated in healthy individuals and are under genetic control. Mutations in key genes that affect hematological parameters have important phenotypic consequences, including multiple variants that affect susceptibility to malarial disease. However, most variation in hematological traits is continuous and is presumably influenced by multiple loci and variants with small phenotypic effects. We used a newly developed mouse resource population, the Collaborative Cross (CC), to identify genetic determinants of hematological parameters. We surveyed the eight founder strains of the CC and performed a mapping study using 131 incipient lines of the CC. Genome scans identified quantitative trait loci for several hematological parameters, including mean red cell volume (Chr 7 and Chr 14), white blood cell count (Chr 18), percent neutrophils/lymphocytes (Chr 11), and monocyte number (Chr 1). We used evolutionary principles and unique bioinformatics resources to reduce the size of candidate intervals and to view functional variation in the context of phylogeny. Many quantitative trait loci regions could be narrowed sufficiently to identify a small number of promising candidate genes. This approach not only expands our knowledge about hematological traits but also demonstrates the unique ability of the CC to elucidate the genetic architecture of complex traits.

Hematological parameters such as red (RBC) and white blood cell (WBC) counts and hemoglobin (Hb) concentration are tightly regulated traits with high clinical relevance. Values outside normal ranges are diagnostic for disorders, including cancer, immune diseases, and cardiovascular disease. In humans, hematological parameters have heritabilities >50% (Evans *et al.* 1999; Lin *et al.* 2007), and mutations in key genes have important phenotypic consequences. Hemoglobinopathies, including the hemoglobin β S allele that plays a role in malarial resistance, are widespread in human populations and are among the most intensively studied human genetic diseases. In recent genome-wide association studies in humans, researchers have identified numerous loci that affect hematological parameters and demonstrated that these traits are subject to complex genetic control (Ferreira *et al.* 2009; Ganesh *et al.* 2009; Gudbjartsson *et al.* 2009; Kamatani *et al.* 2010; Nalls *et al.* 2011; Okada *et al.* 2011; Reiner *et al.* 2011; Soranzo *et al.* 2009; Uda *et al.* 2008). In addition, quantitative trait locus (QTL) mapping studies in mice have identified multiple loci for hematological traits, several of which are homologous to QTL mapped in human studies(Cheung *et al.* 2004; Peters *et al.* 2005, 2006, 2010; Philip *et al.* 2011; Uda *et al.* 2008; Valdar *et al.* 2006).

We sought to identify the genetic determinants of hematological parameters in developing lines of the Collaborative Cross (CC). The CC is a panel of recombinant inbred lines derived from eight-way crosses using inbred mouse strains (Collaborative Cross Consortium 2012). The eight founders of the CC include five classical inbred strains (C57BL/6J, 129/SvImJ, A/J, NOD/ShiLtJ, NZO/HlLtJ) and three wild-derived inbred strains (WSB/EiJ, PWK/PhJ, and CAST/EiJ). The wild-derived strains are representative of three *Mus musculus* subspecies that exist in nature (*domesticus*, *musculus*, *castaneus)*. These eight genomes vary at more than 45 million single nucleotide polymorphisms (SNPs) as well as several million structural variants (Keane *et al.* 2011) and are related through both ancient and recent shared ancestry. Recent genome analysis concluded that classical inbred strains descended from just a few individual mice and are primarily of *domesticus* ancestry (Yang *et al.* 2011). When we consider SNP similarity across the entire genome, the classical inbred strains are very similar to each other, and they are more similar to the WSB/EiJ strain (*domesticus*) than to the PWK/PhJ strain (*musculus*) or CAST/EiJ strain (*castaneus*). However, these relationships vary locally within the genome.

We surveyed hematological parameters in the eight CC founder strains and 131 incipient lines of the CC (pre-CC). Genetic characteristics and QTL mapping strategies for this population have been previously described (Aylor *et al.* 2011; Durrant *et al.* 2011; Philip *et al.* 2011). We identified QTL for mean red cell volume, white blood cells, percent neutrophils/lymphocytes, and monocyte number and evaluated the relative effect of the eight segregating alleles. Because alleles underlying QTL are likely shared between strains, we examined the eight founder genomes for shared ancestry within each QTL interval and identified regions in which the functionally similar alleles were genetically similar. This approach allowed us to narrow QTL regions as much as 10-fold and provided insight into the history of causal variants. Our study demonstrates the power of the CC for complex trait mapping, illustrates the utility of CC-related bioinformatics resources, and provides a strategy for extending genetic analysis beyond QTL.

## Materials and Methods

### Mice

We obtained 131 male pre-CC mice from Oak Ridge National Laboratory. Each mouse was from a distinct CC line that had undergone five to 14 generations of inbreeding. For each CC founder strain, we obtained five to ten male mice from The Jackson Laboratory. All mice were singly housed in the same Association for the Assessment and Accreditation of Laboratory Animal Care−approved facility at National Institutes of Health under normal 12-hr light/dark cycles. Mice were fed NIH-31 Open Formula mouse sterilized diet and allowed to acclimate for a minimum of 1 week before the experiment. To determine the mode of inheritance of *Mcvq4*, we crossed C57BL/6J mice with 129S1/SvImJ mice to generate reciprocal F1 hybrids. Male mice were then phenotyped at 5 to 6 weeks of age.

### Phenotyping

At 10 to 14 weeks of age, blood was drawn from the retro-orbital sinus and analyzed for hematological parameters using a HEMAVET Multispecies Hematology analyzer. The hematology panel (supporting information, Figure S1) included RBCs (no./μl), mean (red) cell volume (MCV, fL), |Hb (g/dL), red cell distribution width (RDW, %), platelets (PLT, no./μl), mean platelet volume (fL), WBCs (no./μl), neutrophils (NE, no./μl), lymphocytes (LY, no./μl), monocytes (MO, no./μl), basophils (no./μl), and eosinophils (no./μl). The number of eosinophils and basophils counted were very small and, therefore, were not used for mapping. We analyzed only the aforementioned parameters and not the derived metrics for hematocrit and mean corpuscular hemoglobin concentration because the derivations are not consistent across strains (S. N. P. Kelada and F. Collins, unpublished data). The following data were log-transformed before analysis: PLT, WBC, LY, MO, and NE. We also examined the WBC data in terms of relative proportions (*i.e.* percent of WBCs). Analysis of variance was used to identify traits that differed between strains. Broad-sense heritability was estimated from the founder lines by calculating the interclass correlations (*r*_1_), the proportion of the total variation accounted for by strain differences, and the coefficients of genetic determination (*g*^2^), which accounts for the doubling of the additive genetic variance with inbreeding, as described previously (Festing 1979).

### Genotyping and QTL mapping

We genotyped each mouse at the University of North Carolina–Chapel Hill, using one of two Affymetrix SNP arrays (A or B) that were produced in development of the Mouse Diversity array (Yang *et al.* 2009). After removing uninformative and poorly performing SNPs, these arrays contained 181,752 (A-array) and 180,976 (B-array) SNP assays, and the set of SNPs on each array did not overlap. Most mice (83%) were genotyped on the B-array and the remaining were genotyped on the A-array. These training arrays were annotated to NCBI Build 36 of the mouse genome, but we mapped QTL boundaries to Build 37 positions to integrate with other resources. We report Build 37 positions in our results. We estimated the most probable ancestor for each SNP in each mouse using the GAIN algorithm (Liu *et al.* 2010) and reconstructed founder haplotypes on the basis of these results. We then merged the nonoverlapping SNP datasets from arrays A and B by imputing unobserved genotypes on the basis of inferred founder haplotype. For QTL mapping, we used HAPPY (Mott *et al.* 2000) to infer ancestry matrices for an additive genetic model. For efficiency, we then averaged the matrices across SNPs between which GAIN inferred no recombination in the population, and this reduced the mapping dataset to 27,059 intervals. We used BAGPIPE (Valdar *et al.* 2009) to fit a regression model and report logarithm of odds (LOD) scores. Significance thresholds were determined by permutation. A 1.5 LOD drop interval is considered the best approximation to a 95% confidence interval in QTL mapping (Dupuis and Siegmund 1999).

### Identification of regions of shared ancestry and phylogenetic analysis

We used a comparative approach to narrow QTL regions and identify candidate genes. Using the allele effect plots as a guide, we grouped founder alleles into two groups by effect (*e.g.* high *vs.* low allele effect). We used the coefficients of the BAGPIPE regression model as allele effects estimates and scaled the eight allele effects relative to the mean of the pre-CC sample. We divided the two groups on the basis of the greatest difference between ordered allele effects estimated at the QTL peak. For *Moq1*, the CAST/EiJ allele effect did not fall clearly into either of the two groups, and we address this separately in the results. We then used the Mouse Phylogeny Viewer at the University of North Carolina (Yang *et al.* 2011) and genome sequences from the Wellcome Trust Sanger mouse genomes project (MGP) (Keane *et al.* 2011; Yalcin *et al.* 2011) to identify regions in the confidence interval in which grouped strains have shared SNP genotypes (positions identified as heterozygous were omitted from the analysis). Regions of shared ancestry were declared where strains share >98% of SNP genotypes. Structural variants may also be important (Yalcin *et al.* 2011) but are outside the scope of our current analysis. Where we could identify regions of shared ancestry, we used MGP SNP data to resolve region boundaries and to generate sequence alignments for downstream phylogenetic analyses. We determined the best model for each alignment using JModelTest (Posada 2009). Then, we used PhyML (Guindon *et al.* 2009) to create maximum likelihood trees. We used the TVMef model for the *Mcvq4* tree, and we used the TVM model for the *Mcvq5* and *Moq1* trees. For each tree, we used PhyML to generate 100 bootstraps (using the corresponding best model). We then used RaxML (Stamatakis 2006) to apply the bootstrap values to each maximum likelihood tree.

### Gene Expression

We evaluated the relationship between hemoglobin β gene (*Hbb*) expression and MCV using quantitative PCR (Table S3). We extracted total RNA from the spleens of 30 pre-CC mice and then synthesized cDNA. We designed the following primers to avoid SNPs that are known in the founder strains of the CC (Keane *et al.* 2011):

*Hbb-total* F1: GACCTATCCTCTGCCTCTGC;
*Hbb-total* R1: ATCCACATGCAGCTTGTCAC;
*Hbb-b1* F2: ACCTGGGCAAGGATTTCAC;
*Hbb-b1* R2: AGCTAGATGCCCAAAGGTCTTC;
*Rps29* F: GGAGTCACCCACGGAAGT;
*Rps29* R: TCCATTCAAGGTCGCTTAGTC.

We validated all primer pairs for specificity by sequencing and amplification efficiency using serial dilutions of template. We chose *Rps29* as the control on the basis of its suitability as a reference (De Jonge *et al.* 2007). We examined gene expression data in two ways: (1) we calculated mean expression using the ΔCt method (relative to *Rps29*), and (2) we built a linear regression model for MCV including terms for *Hbb* gene expression (total or Hbb-b1) expressed as the ΔCt, and *Hbb s vs. d* genotype. To facilitate interpretation of these results, we plotted MCV *vs.* –ΔCt so that higher gene expression corresponds with larger *x*-axis values.

## Results

**Figure 1:**
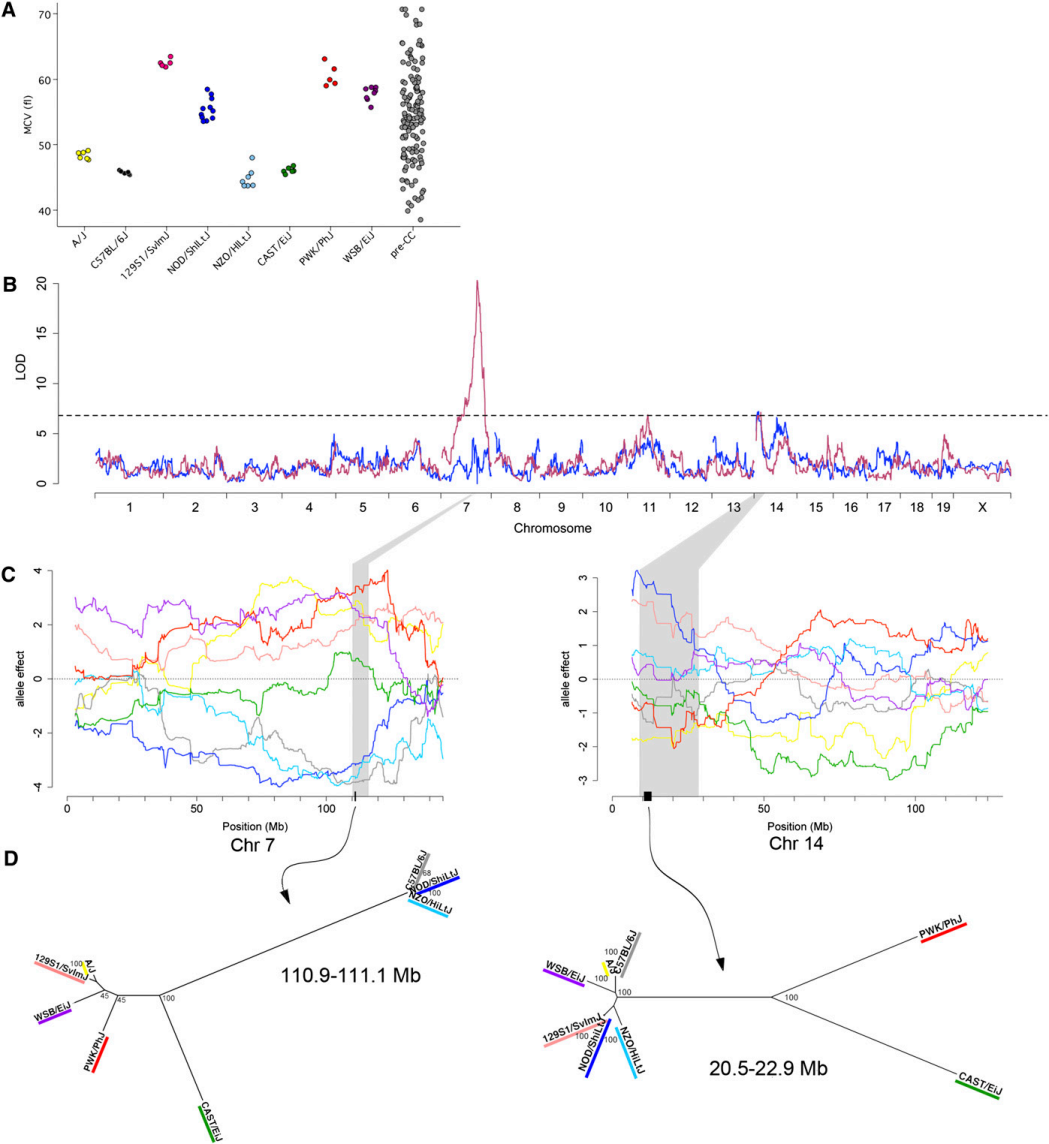
(A) Distribution of MCV in CC founder strains and pre-CC mice. (B) Genome scan for MCV (maroon) and genome scan conditioned on *Hbb* genotype (blue). The dashed line denotes 95% significance threshold determined by permutation. (C) Allele effects plots for *Mcvq4* on Chr 7 (left) and *Mcvq5* on Chr 14 (right). Shaded areas correspond to the QTL confidence interval, and the dark bars on the x-axis denote the regions identified by analysis of shared ancestry as described in text. Color-coding of allele effects is as follows: dark gray, C57BL/6J; light blue, NZO/HlLtJ; blue, NOD/ShiLtJ; green, CAST/EiJ; yellow, A/J; salmon, 129S1/SvImJ; red, PWK/PhJ; purple, WSB/EiJ. (D) Phylogenetic trees for regions of shared ancestry in *Mcvq4* (left) and *Mcvq5* (right). Trees were constructed using SNP data from Sanger MGP (see *Materials and Methods*), and the numbers indicate bootstrap support estimates for each branch.

**Figure 2:**
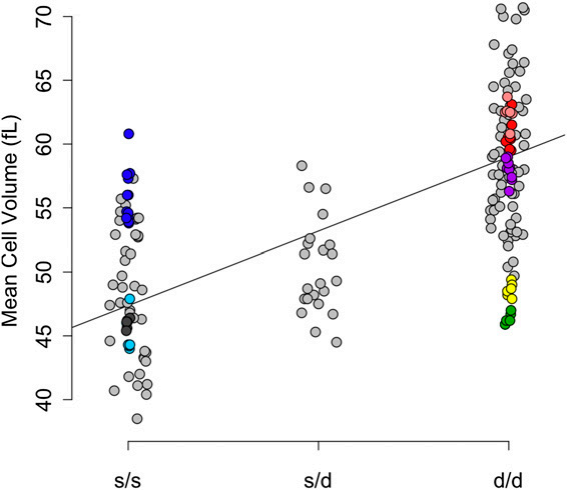
MCV as a function of *Hbb* genotype in pre-CC mice (light gray) and founder strains. The color scheme is the same as that used in Figure 1. The regression line shows the effect of the *s vs*. the *d* allele. Heterozygotes showed intermediate phenotypes.

**Figure 3:**
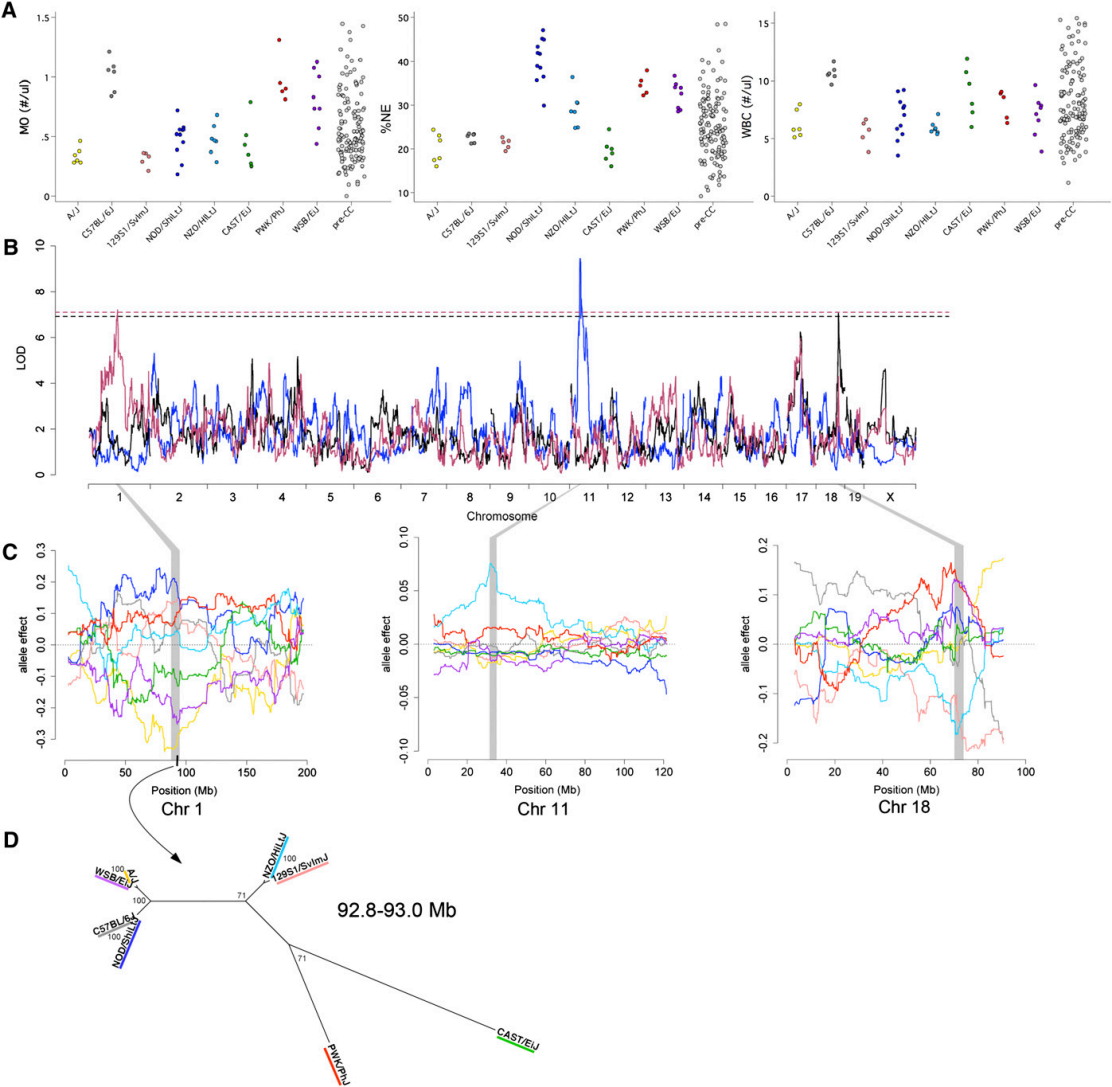
(A) Distributions of MO, %NE, and WBC. (B) Genome scans for MO (maroon), %NE (blue), and WBC (black). Dashed lines represents 95% significance threshold determined by permutation for MO (maroon), %NE and WBC (black). (C) QTL allele effects plots for *Moq1* (left), *NE_pct1* (center), and *Wbcq7* (right). The color scheme is the same as that used in Figure 1. For *Moq1*, the CAST/EiJ allele effect (green) is intermediate to the low-MO and the high-MO group. Shaded areas correspond to the QTL confidence interval, and the dark bar on the x-axis for *Moq1* denotes the region identified by analysis of shared ancestry as described in (D). Phylogenetic tree for the region in *Moq1*of shared ancestry among the low-MO group, spanning 92.8 to 93.0 Mb on Chr 1.

We surveyed RBC and WBC parameters of the eight founder lines and 131 male pre-CC mice to determine the amount of phenotypic variation present in the incipient lines of the CC. The distributions of each trait are shown in Figures 1−3 and Figure S1, and summary statistics for each parameter are shown in Table S4. Using these data, we identified five QTL for four hematological parameters: MCV, WBC, MO, and percent NE. We did not identify QTL for RBC, Hb, RDW, PLT, or mean platelet volume.

### MCV

Evidence for a strong genetic component to variation in MCV was readily apparent from the eight founder strains (Figure 1A, *F*-test, *P* < 1.0 × 10^−4^). The distribution of MCV among pre-CC mice both exceeded and interpolated the values of the eight founder strains. These results suggested a polygenic basis of inheritance.

We used a single-QTL model to map a large-effect QTL on Chr 7 (*Mcvq4*, 110.3−116.2 Mb, LOD = 20.3, Figure 1B and Table 1) and two additional QTL on Chr 11 (53.4-61.7 Mb, LOD = 6.9) and Chr 14 (8.9−22.9 Mb, LOD = 7.1). Conditioning on *Mcvq4* resulted in the Chr 11 QTL falling below a 95% genome-wide significance threshold, but the Chr 14 QTL was largely unaffected (*Mcvq5*, LOD = 7.2) and a new peak on Chr 14 crossed a 90% suggestive threshold (63.7−77.0 Mb, LOD = 6.6).

**Table 1:** Summary of significant QTL identified for hematologic parameters

Trait	Name	Chr	Peak, Mb	LOD	95% CI Start, Mb	95% CI End, Mb	Size, Mb	No. Genes	Candidate Interval Size, Mb	No. Genes after Reduction of CI
MCV	*Mcvq4*	7	111.815	20.3	110.336	116.242	5.906	227	0.127	10
MCV	*Mcvq5*	14	15.659	7.1	8.852	22.898	14.046	93	2.445	37
WBC	*Wbcq7*	18	70.655	7.1	69.996	74.124	4.128	18	NA	NA
% Neutrophils^a^	*Ne_pctq1*	11	32.894	9.5	31.296	35.192	3.774	48	NA	NA
Monocytes	*Moq1*	1	92.817	7.2	87.560	94.962	7.402	49	0.167	4

QTL, quantitative trait locus; LOD, logarithm of odds; CI, confidence interval.

a % Lymphocytes maps to the exact same location but with a lower LOD score.

*Mcvq4* included the well-studied hemoglobin β (*Hbb*) locus that has a demonstrated effect on MCV (Kano and Nishida 1972), and the estimated QTL allele effects (Figure 1C) matched the previously reported strain assignments of the *Hbb single* (*s*; C57BL/6J, NOD/ShiLtJ, and NZO/H1LtJ) and *diffuse* (*d*; 129S1/SvImJ, A/J, Cast/EiJ, PWK/PhJ and WSB/EiJ) alleles (Erhart *et al.* 1985; Gilman 1976; Popp *et al.* 1982). The haplotypes associated with both alleles include a duplicated *Hbb* gene, with the two genes denoted *Hbb-b1* and *Hbb-b2*. In the *s* haplotype, the *Hbb-b1* and *Hbb-b2* genes code for identical protein products. In the *d* haplotype, those genes code for two different functional β-globins. The protein products of *Hbb-b1* and *Hbb-b2* are referred to as the major and minor chains, respectively, because *Hbb-b1* is expressed at greater levels than *Hbb-b2*. Table S5 shows the amino acid positions that distinguish the protein products of the *s* and *d* alleles.

To compare the effect of *Mcvq4* between the founder strains and the pre-CC population, we categorized all pre-CC mice by *Hbb* genotype (*s/s*, *s/d*, or *d/d*) on the basis of their inferred founder haplotype across that region and the previously reported *Hbb* alleles for each founder strain. Mice with *s*/*s* genotype had lower MCV than *d*/*d* mice on average in both the founder strains (*F*-test, *P* ≤ 7.8 × 10^−4^) and the pre-CC mice (*F*-test, *P* < 1.0 × 10^−4^). Heterozygous *s/d* genotypes were intermediate (Figure 2). We confirmed this partial dominance in C57BL/6J (*s/s* genotype) × 129S1/SvImJ (*d/d* genotype) F1 hybrid male mice that also had intermediate MCV values (Figure S2). In addition, we noted a modest but significant difference in MCV between F1 hybrid mice from 129S1/SvImJ dams and F1 hybrid mice from the reciprocal cross (55.6 ± 0.4 *vs.* 53.4 ± 0.4 fL, respectively, *P* = 0.002), which could be attributed to X-linked inheritance or a parent-of-origin effect. There were also differences in the estimated effects of the *Hbb* alleles between the two populations. Specifically, *Hbb* genotype explained 49% of MCV variance in the pre-CC population but only 17% of the variance in the eight founder strains. Plotting the founder strain phenotypes by *Hbb* genotype sheds light on this discrepancy: CAST/EiJ and A/J both fall well below the *d/d* group average, whereas NOD/ShiLtJ is well above the *s/s* group average (Figure 2).

In an analysis of variance of the founder lines considering both *Hbb* genotype and strain, strain explained nearly 80% of the variance independent of the allele effect. This finding suggests that the aberrant strains either have unique *Hbb* alleles (neither *s* nor *d*) or harbor additional genetic factors affecting MCV. To test whether these strain differences resulted from previously unreported *Hbb* alleles, we compared the average MCV for pre-CC mice with each founder haplotype to the average MCV for each founder strain (Table S6). Generally, pre-CC mice regressed to the MCV mean for the *s/s* or *d/d* genotype class, not the MCV mean for that founder strain. For example, pre-CC mice that were homozygous for CAST/EiJ alleles had an average MCV of 56.82 fL, close to the *d/d* group average of 59.45 fL, whereas MCV among CAST/EiJ founder mice was 46.32 fL. These results suggest that the *Hbb* locus was biallelic and one or more additional MCV loci explained the deviation of inbred strains from their *Hbb* allele group average.

On the basis of our conclusion that two functional *Hbb* alleles were segregating in the pre-CC population, we used a comparative approach to identify regions of shared ancestry within *Mcvq4*. We grouped founder strains by their *Hbb* alleles and then analyzed Sanger MGP SNP data (Keane *et al.* 2011; Yalcin *et al.* 2011) to identify regions in the QTL confidence interval for which the strains within each group were genetically identical. We identified a 127-kb region (Chr7: 110,959,072-111,086,522 bp, Table S7 and Table S8) containing *Hbb-b1 and Hbb-b2*, three other *Hbb* genes, and five olfactory receptor genes. The three *s/s* strains were identical at 99% of the 3499 SNPs in the region and did not share ancestry with any of the *d/d* strains across this region, whereas the *d/d* strains shared only 31% identity. This analysis supported our conclusion that *Hbb* is the causal locus underlying *Mcvq4*, and suggested that the *s* allele arose more recently than the *d* allele.

A recent report gave evidence of differences in *Hbb* gene expression by s/*s vs. d/d* genotype (Peters *et al.* 2010). Using spleen RNA from a subset of pre-CC mice (n = 30), we compared mean *Hbb-b1* gene expression in *s/s* (ΔCt = −2.0 ± 1.4) *vs. d/d* (ΔCt = −1.1 ± 1.5) mice. The high variance within groups meant the difference in means was not statistically significant (*P* = 0.13). We also examined whether expression of *Hbb-b1* correlated with MCV and found that *Hbb-b1* expression was not associated with MCV in a model that did not account for genotype. However, a linear trend was noticeable within each genotype class (*P* = 0.08 for *Hbb-b1* expression term, Figure S3), and this relationship was similar across genotypes (*i.e.* there were no interactions between genotype and expression, Table S9).

We asked whether the QTL on Chr 14, *Mcvq5*, could explain some of the additional MCV differences that we observed between founder strains. The effects the NOD/ShiLtJ and 129S1/SvImJ alleles were associated with the greatest MCV, whereas there was no clear separation among the strains associated with the lowest MCV (Figure 1C). We performed an analysis of shared ancestry on the initial 14.0-Mb confidence interval and identified a region spanning 20,453,181 to 22,963,216 bp where NOD/ShiLtJ and 129S1/SvImJ share 99% identity (Table S7). The phylogenetic tree for this region (Figure 1D) shows that the low-MCV alleles are highly diverged, with the wild-derived CAST/EiJ and PWK/PhJ alleles distant from all the classical inbred strains and WSB/EiJ. This finding suggests that the high-MCV allele was derived from a low-MCV allele. We restricted our subsequent analysis to the 2.5-Mb region shared by the two high-MCV alleles, which contains 37 genes (Table S8). We used gene ontology and molecular phenotype annotations to ask if any of the 37 genes had been associated with hematological phenotypes previously. Annexin A7 (*Anxa7*) is in the middle of the region, is known to be expressed in erythrocytes, and *Anxa7* knockout mice have increased MCV (Herr *et al.* 2003). We propose that *Anxa7* is a plausible candidate gene for *Mcvq5*.

### WBC parameters

We examined the distributions of total WBCs and WBC subpopulations of LY, NE, MO, BA, and EO (Figure 3A and Figure S1). For the majority of mice tested, the numbers of BA and EO counted were very small (averages of 0.3% and 1.3% of WBC, respectively) and hence these two traits were not pursued further. LY, NE, and MO comprised 66.3%, 25.0%, and 7.1% of WBCs, respectively. Predictably, there were strong positive correlations between the numbers of these three WBC populations (*r* = 0.71 for MO and NE, 0.60 for LY and NE, and 0.57 for LY and MO). The range of WBC, LY, and NE among pre-CC mice exceeded those of the founder strains at the upper end of the spectrum (Figure 3A and Figure S1).

We identified a significant QTL for WBC on Chr 18 (*Wbcq7*, 70.0-74.1 Mb, LOD = 7.1) (Figure 3A and Table 1). We detected suggestive peaks (*P* < 0.2) for LY and NE at the same locus, which is explained by their large contributions to total WBC counts. 129S1/SvImJ and NZO/HlLtJ alleles were in the low-WBC group and were clearly separate from the other strains (Figure 3C). These two founder strains also had lower WBC on average, but the within-strain variance was high and the differences between strains were not significant. The ancestral haplotype structure in this region of the genome shows that all eight strains have unique patterns except A/J and NOD/ShiLtJ, which are nearly identical to each other. Although there are broad regions in which the 129S1/SvImJ and NZO/HlLtJ strains are identical, in most of these regions these strains are also identical to one of the strains from the high-WBC group. We searched for regions in which the 129S1/SvImJ and NZO/HlLtJ strains share haplotypes and are also distinct from other strains. Only 19 SNPs across the 4.1-Mb region met these criteria. Eleven of these SNPs were in introns of the gene *Dcc*, making it a strong candidate gene (Table S8). Another plausible explanation for the allele effects pattern is that the 129S1/SvImJ and NZO/HlLtJ genomes have different polymorphisms that lead to the same effect.

We evaluated NE and LY in terms of relative proportions (percent). Percent NE and percent LY have a strong negative correlation (*r* = –0.94). Therefore, these measures essentially reflect one trait describing WBC composition that we refer to as %NE for simplicity. Four strains showed low %NE (A/J, C57BL/6J, 129S1/SvImJ, CAST/EiJ) of 19.9% to 22.8%, whereas the NOD/ShiLtJ strain averaged 40.4% NE. The NZO/HlLtJ, PWK/PhJ, and WSB/EiJ strains showed intermediate %NE (Figure 3A). We found a QTL (*NE_pctq1,* Chr 11: 31.3-35.1 Mb, LOD = 9.45; Figure 3B) for which the NZO/HlLtJ allele effect was distinct from all other allele effects, and was associated with a greater %NE (Figure 3C). A total of 1484 SNPs in the confidence interval were private to the NZO/HlLtJ allele, and they were distributed relatively uniformly across the interval. Since we could not reduce our candidate interval based only on SNP data, we used Gene Ontology and Mammalian Phenotype Ontology to find candidate genes with annotations related to WBC counts and then looked for the NZO/HlLtJ private SNPs in those genes (Table S8). Of the 48 genes in the region, 6 were annotated with WBC-related terms: *Dock2*, *Stk10*, *Il9r*, *Mpg, Lcp2*, and *Npm1*. A total of 292 (4%) of the 7312 SNPs in the *Dock2* gene are private to the NZO/HlLtJ allele, including a nonsynonymous coding polymorphism. *Stk10* had 47 (3.4%) private SNPs, including a nonsynonymous coding polymorphism, *Mpg* and *Lcp2* each had one private SNP (1.6% and 0.1%, respectively), and *Il9r* had 34 (9.7%) private SNPs. In contrast, *Npm1* has no variation that is private to NZO/HlLtJ, so we eliminated it as a candidate gene.

Finally, we mapped a QTL for MO on Chr 1 (*Moq1*, 87.6-95.0 Mb, LOD = 7.20, Figure 3B and Table 1). The A/J and WSB/EiJ alleles were clearly associated with low monocyte count (Figure 3C). The CAST/EiJ allele effect was ambiguous—it was slightly more similar to the A/J and CAST/EiJ alleles at the peak of the QTL, but it was more similar to the high-allele group at the end of the QTL interval. We searched for shared ancestry regions with CAST/EiJ included alternately in each group, but CAST/EiJ did not share regions of ancestry with either group. A/J and WSB/EiJ share a common haplotype from 92,803,891 to 92,970,550 bp that differs from all the other strains at nine SNPs (Figure 3D and Table S7). This region contains four genes (Table S8), and of these four, *Lrrfip1* is the only gene with previous evidence of being expressed in monocytes and having a role in monocyte response to pathogens (Arakawa *et al.* 2010; Yang *et al.* 2010), rendering it the best candidate.

## Discussion

Genetics influences the variation of hematological parameters in multiple species (Chen and Harrison 2002; Cheung *et al.* 2004; Ganesh *et al.* 2009; Peters *et al.* 2005, 2006, 2010; Uda *et al.* 2008; Zou *et al.* 2008). Recent human genome wide association studies have reported strong associations between specific loci and multiple hematological parameters (Ferreira *et al.* 2009; Ganesh *et al.* 2009; Gudbjartsson *et al.* 2009; Kamatani *et al.* 2010; Nalls *et al.* 2011; Okada *et al.* 2011; Reiner *et al.* 2011; Soranzo *et al.* 2009; Uda *et al.* 2008), and in some cases the same loci are also associated with disease risk. For example, SNPs associated with hematocrit (Ganesh *et al.* 2009) are also associated with high blood pressure and hypertension (Levy *et al.* 2009). Likewise, SNPs associated with eosinophil number are associated with the risk of asthma (Gudbjartsson *et al.* 2009). Whether the relationships between the hematologic parameter and disease risk is causal has not yet been determined in many cases. Our findings will allow investigators to choose CC strains with divergent hematological phenotypes and known genotypes as a way to investigate the underlying mechanisms of disease and determine whether these relationships cause disease.

We characterized two previously reported QTL for MCV (D'Surney and Popp 1992) and identified new QTL for WBC, MO, and percent NE by using a relatively small number of pre-CC mice. In addition, we phenotyped founder strain mice and used those results to strengthen our conclusions. The founder strain data gave us an estimate of within-strain variance for each trait. High variance can indicate that a phenotype has low heritability or that measurement error is high, and can complicate QTL mapping. MCV was extremely consistent within strain and resulted in the most significant QTL. After discovering each QTL, we took advantage of the shared ancestry between the CC founder strains to narrow the standard confidence intervals dramatically and to identify candidate regions ranging in size from hundreds of kilobases to a few megabases. This approach not only brought us much closer to identifying causal genes than most QTL studies, but yielded additional insight into the phylogenetic history of the causal alleles.

Our most statistically significant finding confirmed *Hbb* as the causal locus for *Mcvq4. Mcvq4* is coincident with a red cell hemoglobin concentration mean QTL reported in F2 intercrosses (Peters *et al.* 2010), a mean cellular hemoglobin QTL reported in heterogeneous stock (HS) mice (Valdar *et al.* 2006), and a RDW QTL reported in a different set of CC-related mice (Philip *et al.* 2011), indicating that *Hbb* affects multiple RBC traits. The HS QTL was recently attributed to a genomic insertion found in the C57BL/6J strain (Yalcin *et al.* 2011), but this scenario is unlikely because the other *s/s* strains in our study do not share the same insertion. Ten amino acid substitutions distinguish the protein products of the *s vs. d* alleles, and previous work proposed that these affect MCV (D'Surney and Popp 1992). In particular, the G13C and A139T substitutions have the potential to affect protein stability and alter hemoglobin oxygen-affinity, and these substitutions are shared across all *d/d* founder strains. More recently it was proposed that the genetic variation alters MCV through *Hbb* gene expression (Peters *et al.* 2010). In a subset of our sample, we found that variation in *Hbb-b1* gene expression was not correlated with genotype, but was weakly correlated with MCV independent of genotype (Figure S3). We cannot determine whether greater expression increases MCV, or if increased MCV leads to greater *Hbb* expression.

The simplest explanation for the presence of all three wild-derived strains in the *d/d* group, the genetic distance between the two groups, and the identity within the *s/s* group, is that *d* is the ancestral allele and that *s* arose in classical inbred strains long enough ago to accumulate *de novo* mutations (*n* = 3 SNPs). However, recent work in the field shows that both *Hbb* alleles are present in wild mouse populations and suggests that these alleles are maintained by balancing selection (Storz *et al.* 2007). The same study did not support altitude adaptation as a selective pressure on *Hbb*, though the two alleles show differences in oxygen binding affinity (D'Surney and Popp 1992; Newton and Peters 1983). Another hypothesis is that allelic differences in the redox status of hemoglobin and glutathione may affect response to parasites such as murine *Plasmodium* species, which are known to be sensitive to RBC redox status (Becker *et al.* 2004). Whatever the selective pressures on *Hbb,* this evidence suggests that the *s* and *d* alleles were independently contributed to the classical inbred strains from their wild ancestors. Furthermore, recent results show that both alleles segregate in all three *Mus* subspecies (John Didion and F. Pardo-Manuel de Villena, unpublished data). Therefore, it is likely just chance that the three wild-derived CC founder strains share the *d* allele.

A linear model relating *Hbb* genotype to MCV fit the pre-CC data better than it fit the founder strain data, and led to two different heritability estimates. Because the same alleles are in both populations, we would expect to observe similar heritability. This paradox illustrates an important point about estimating heritability in structured populations. We expected that the small heritability in the founder strains resulted from undetected QTL in strains such CAST/EiJ and A/J, which had MCV well below the mean of the *Hbb d/d* group. Because these genetic background effects are correlated with *d/d* genotypes in those strains, it had the effect of lowering the heritability estimate. In the CC, the genetic background is randomized through the process of creating recombinant inbred lines (Churchill *et al.* 2004). We showed that pre-CC MCV regressed to the mean for the *Hbb* genotype, not the mean for the inbred strain from which the allele was inherited (Table S5). This demonstrates the power of QTL mapping in randomized backgrounds and illustrates the shortcomings of *in silico* mapping that have been discussed elsewhere (Collaborative Cross Consortium 2012; Payseur and Place 2007)

Mice with 129S1/SvImJ and NOD/ShiLtJ alleles had high MCV relative to the rest of their *Mcvq4* allele groups. The allele effects at *Mcvq5* may help explain this observation, since those two alleles are associated with increased MCV (Figure 2). We propose that *Anxa7* is a highly plausible candidate gene because it lies in a region of shared ancestry, it is expressed in RBCs, and has a known role in RBC physiology. Furthermore, *Anxa7* knockout mice show increased MCV (Herr *et al.* 2003) as well as differential response to osmotic stress (Lang *et al.* 2010). An MCV QTL found in HS mice (20.7−21.5 Mb) (Valdar *et al.* 2006) is consistent with *Mcvq5* and *Anxa7*. The additional suggestive peak that we found on Chr 14 (Figure 1B) is consistent with the *Chcmq14* QTL reported by Peters *et al.* (Peters *et al.* 2010) and an MCV QTL reported in HS mice (64.7−65.1 Mb) (Valdar *et al.* 2006). We did not detect the previously reported QTL *Chcmq*2, possibly because of power considerations, or *Chcmq8*, which was female-specific.

*Wbcq7* appears to be driven by primarily by lymphocytes, which comprise the largest fraction of WBCs. The low-WBC alleles shared multiple SNPs in the first intron of *Dcc*, which includes regions that show regulatory potential and are conserved across mammals (UCSC Genome Browser). *Wbcq7* overlaps with four previously reported mouse QTL related to immune system phenotypes: insulin-dependent diabetes susceptibility (*Idd21.2*) (Hollis-Moffatt *et al.* 2005), pulmonary adenoma resistance and pulmonary adenoma susceptibility (*Pas2* and *Pas7*) (Festing *et al.* 1998; Malkinson *et al.* 2002), and proteoglycan induced spondylitis 1 (*Pgis1*) (Vegvari *et al.* 2005). *Dock2* is perhaps the most intriguing of the six candidates we identified for *Ne_pctq1* because it is expressed in hematopoetic cells and *Dock2* knockout mice have altered LY and NE responses to chemokines (Fukui *et al.* 2001). We narrowed *Moq1* to a 167-kb region that includes *Lrrfip1*, a gene known to be involved in MO function (Arakawa *et al.* 2010; Yang *et al.* 2010).

We used a shared ancestry approach for narrowing QTL confidence intervals on the basis of two assumptions. First, we assumed two functional alleles were present, and we grouped the eight founder alleles by their effect on each phenotype. Our method for grouping alleles is simple. We relied on the differences between the eight effects estimates, refined by our observation of the allele effects plots (Figures 1C and 3C). For four QTL the effects were easily divided into two groups, but the CAST/EiJ effect for *Moq1* was ambiguous and we considered it a third allele. This illustrates a weakness of our approach: there is no formal way for finding the number of alleles or the correct grouping of alleles. New methods that are in development should remedy these concerns, and better address heterozygosity. Second, we assumed that a common polymorphism separated the functional groups. Classical inbred mouse strains are descended from a small number of progenitors (Yang *et al.* 2011), and their recent shared ancestry makes it unlikely that multiple functional variants arose in a single QTL. Nonetheless, we cannot rule out multiple functional alleles. This method is similar in spirit to merge analysis (Yalcin *et al.* 2005), which also uses genetic similarity to narrow QTL regions in multi-founder crosses. Merge analysis tests if the association between a trait and a SNP genotype exceeds the significance of an association using a multi-allele model. However, both methods are subject to the same basic assumptions.

We emphasize that shared ancestry analysis of the CC founders is independent from statistical mapping resolution. We reported five QTL confidence intervals (on the basis of 1.5 LOD decrease) that ranged from 3.8 to 14.0 Mb, which is excellent for a mouse QTL study. Shared ancestry substantially narrowed three of these (*Mcvq4*, *Mcvq5*, *Moq1*), by 98%, 83%, and 98%, respectively. The resulting candidate intervals are much narrower than those that have previously been reported in mice. The CC was not designed for optimal mapping resolution. Increased resolution would have required a different breeding design with additional generations of recombination (Svenson *et al.* 2012), and such a design would risk introducing population structure. The significance of our approach is that the additional information from ancestral recombination more than offsets any loss of resolution relative to another design, at least in these three cases. It is instructive to consider why we were unable to narrow our interval using shared ancestry for the other two QTL, *Wbcq7* and *NE_pctq1*. For each successful QTL, only one group had clear regions of shared ancestry and in two cases that group included only classical inbred strain alleles. Because wild-derived strains are so genetically distant from the others, it is less likely that they will share functional polymorphisms with classical inbred strains. The low-MO group at *Moq1* was an exception and included the wild-derived WSB/EiJ strain, but WSB/EiJ is phylogenetically closer to the classical inbred strains than CAST/EiJ or PWK/PhJ. Though the low-WBC group for *Wbcq7* consisted of only two classical inbred strains, there were few uniquely shared SNPs. One of these SNPs could be functional, but an equally good explanation is that the two strains harbor separate functional SNPs, or that we assigned the alleles to the wrong groups. We concluded that *NE_pctq1* was unique to the NZO/HlLtJ allele, so there was no shared ancestry to evaluate. However, this is an ideal case for identifying causal variants. If the QTL is true, the effects estimate is true, and the sequence data are error-free, one of the observed private SNPs must be the causal variant for the QTL.

In summary, we have characterized the broad phenotypic diversity of hematological phenotypes in pre-CC mice, identified QTL for several parameters, and used private SNPs and shared ancestry to narrow QTL regions and implicate candidate genes. We conclude that shared ancestry is an extremely powerful approach for fine mapping in the CC.

## Supplementary Material

Supporting Information
