# Supplementary material for: Genetic Analysis of Hematological Parameters in Incipient Lines of the Collaborative Cross
Source: G3 (Bethesda). 2012 Feb 1;2(2):157–65. doi: 10.1534/g3.111.001776 (PMC3284323; doi:10.1534/g3.111.001776)
Supplement: Supporting Information [file supp_2_2_157__index.html]

Supporting Information 

# Genetic Analysis of Hematological Parameters in Incipient Lines of the Collaborative Cross

## Supporting Information for Kelada *et al.*, 2012

**Files in this Data Supplement:**

- Supporting Information - Figures S1-S3 and Tables S1-S9 (PDF, 1.4 MB)
- Figure S1 - Distributions of hematological traits in pre-CC mice and founder lines for which no QTL were detected (PDF, 850 KB)
- Figure S2 - MCV values among C57BL/6J (*Hbb s/s* genotype), 129S1/SvImJ (*Hbb d/d* genotype), and F1 mice (*s/d* genotype) (PDF, 56 KB)
- Figure S3 - *Hbb* gene expression and MCV in pre-CC mice (PDF, 143 KB)
- Table S4 - RBC and WBC Parameters in Collaborative Cross Founder Strain and pre-CC Mice (PDF, 48 KB)
- Table S5 - SNPs in the Hemoglobin β Locus that Distinguish Single vs. Diffuse Strains (PDF, 46 KB)
- Table S6 - MCV (fL) Among Pre-CC mice and Founder Strains as a Function of *Hbb-b1/b2 Single* versus *Diffuse* Genotypes (PDF, 49 KB)
- Table S7 - Regions of Shared Ancestry Identified Using Mouse Genomes Project SNP Data (PDF, 43 KB)
- Table S9 - Regression Models of MCV as a Function of Hbb s/d Genotype and Hbb Gene Expression (PDF, 42 KB)
- Table S1 - (.txt, 17 KB)
- Table S2 - (.zip, 1 KB)
- Table S3 - (.txt, 1 KB)
- Table S8 - (.txt, 143 KB)
