# Supplementary material for: Genetic Analysis of Hematological Parameters in Incipient Lines of the Collaborative Cross
Source: G3 (Bethesda). 2012 Feb 1;2(2):157–65. doi: 10.1534/g3.111.001776 (PMC3284323; doi:10.1534/g3.111.001776)
Supplement: Supporting Information [file supp_2.2.157_001776SI.pdf]

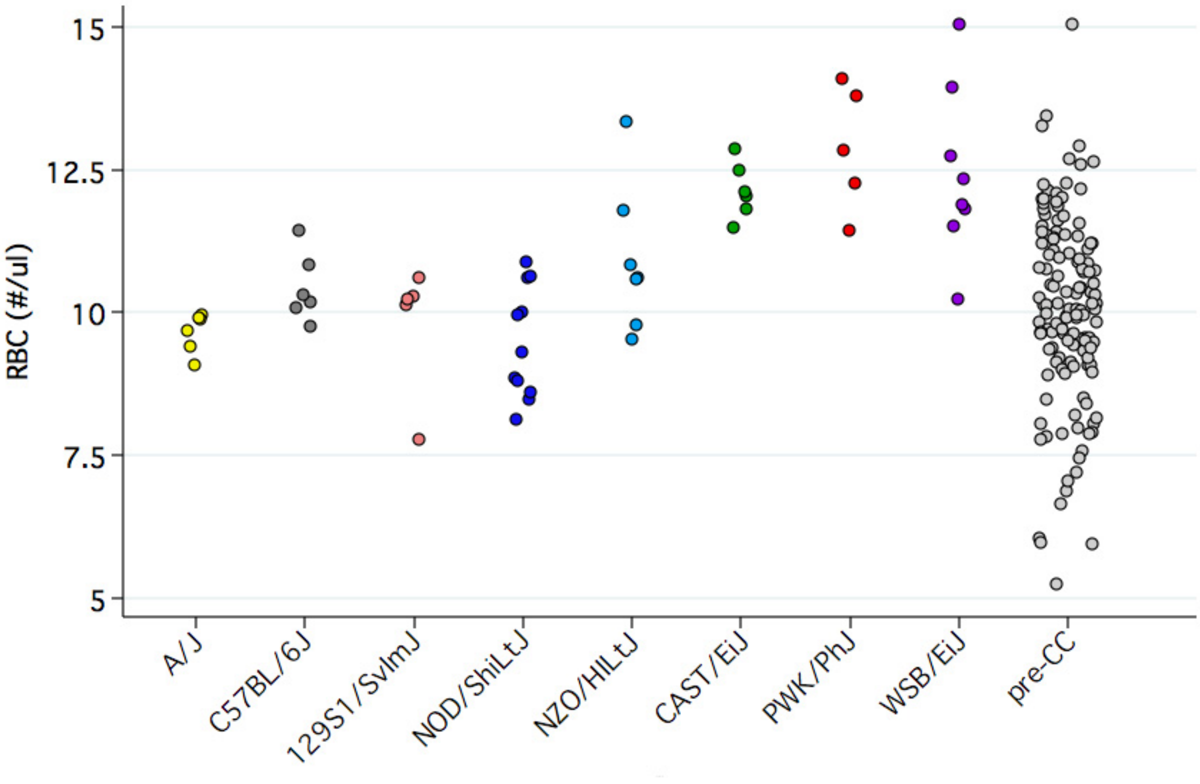

RBC (#/ul)
15
12.5
10
7.5
5
A/J
C57BL/6J
129S1/SvImJ
NOD/ShiLtJ
NZO/HlLtJ
CAST/EiJ
PWK/PhJ
WSB/EiJ
pre-CC

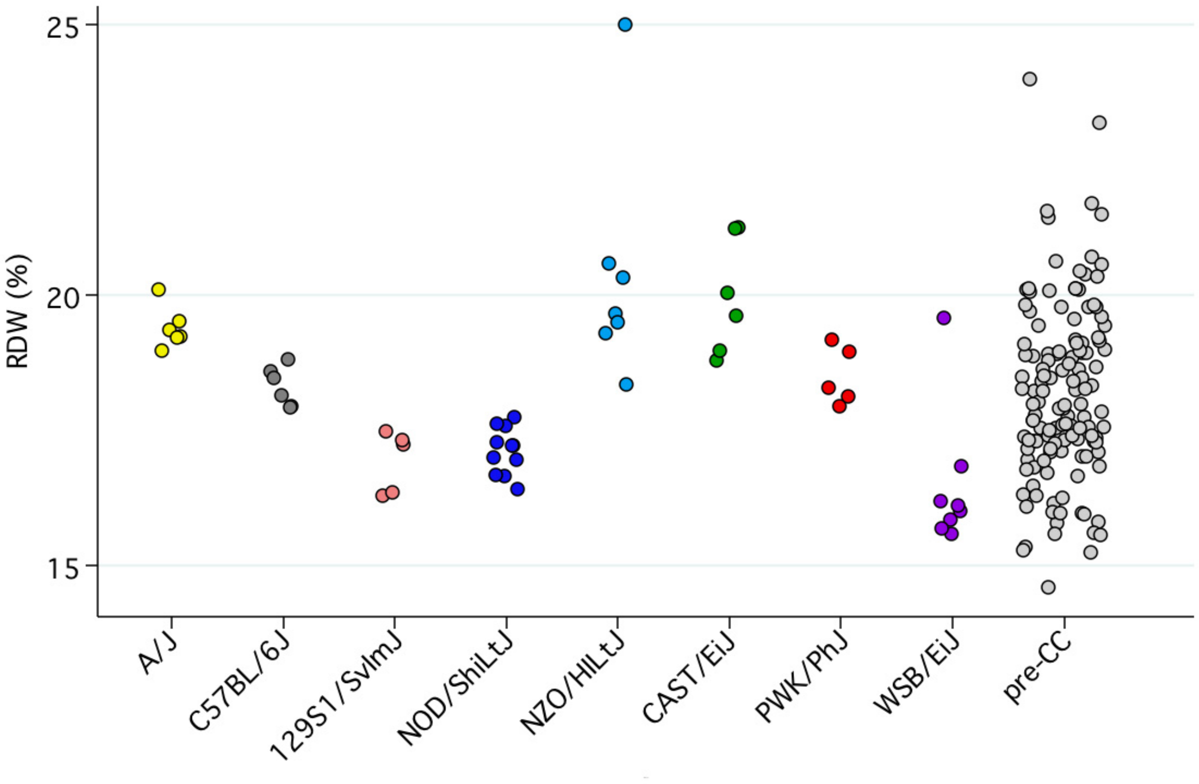

RDW (%)
25
20
15
A/J
C57BL/6J
129S1/SvImJ
NOD/ShiLtJ
NZO/HlLtJ
CAST/EiJ
PWK/PhJ
WSB/EiJ
pre-CC

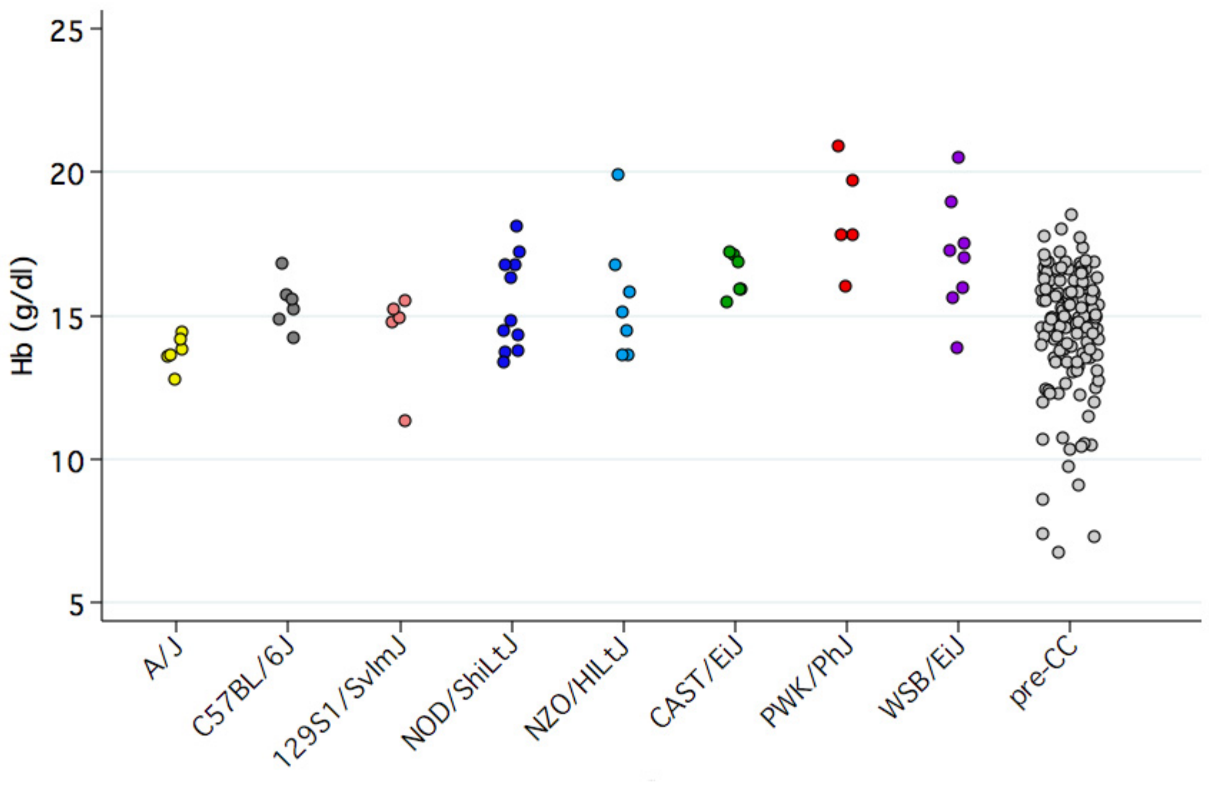
Hb (g/dl)
25
20
15
10
5
A/J
C57BL/6J
129S1/SvImJ
NOD/ShiLtJ
NZO/HILtJ
CAST/EiJ
PWK/PhJ
WSB/EiJ
pre-CC

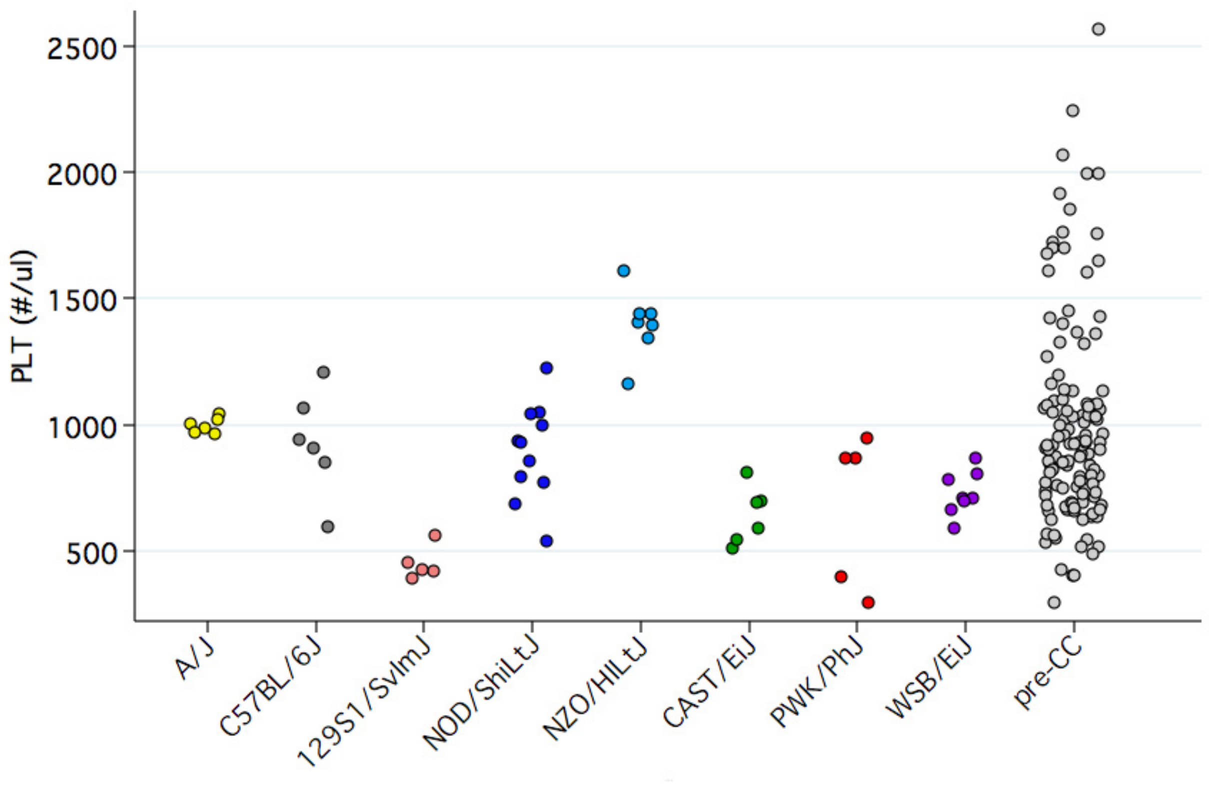
PLT (#/ul)
2500
2000
1500
1000
500
A/J
C57BL/6J
129S1/SvImJ
NOD/ShiLtJ
NZO/HILtJ
CAST/EiJ
PWK/PhJ
WSB/EiJ
pre-CC

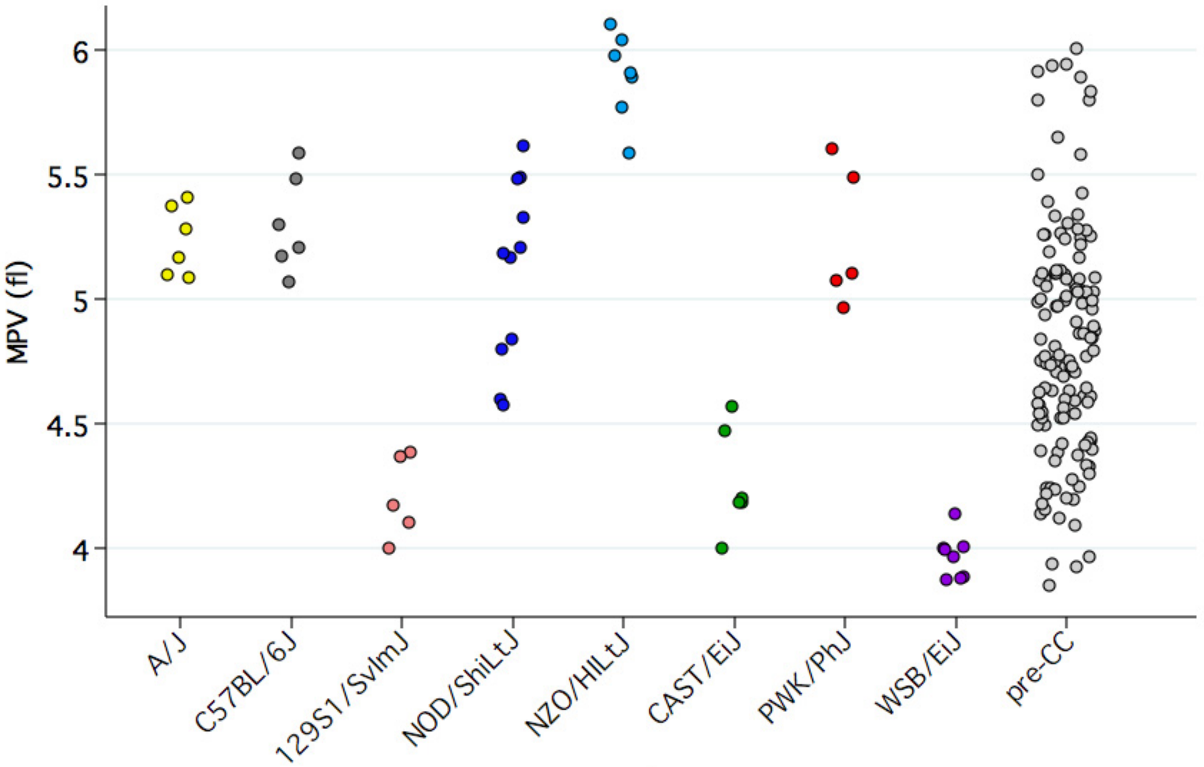
MPV (fl)
6
5.5
5
4.5
4
A/J
C57BL/6J
129S1/SvImJ
NOD/ShiLtJ
NZO/HILtJ
CAST/EiJ
PWK/PhJ
WSB/EiJ
pre-CC

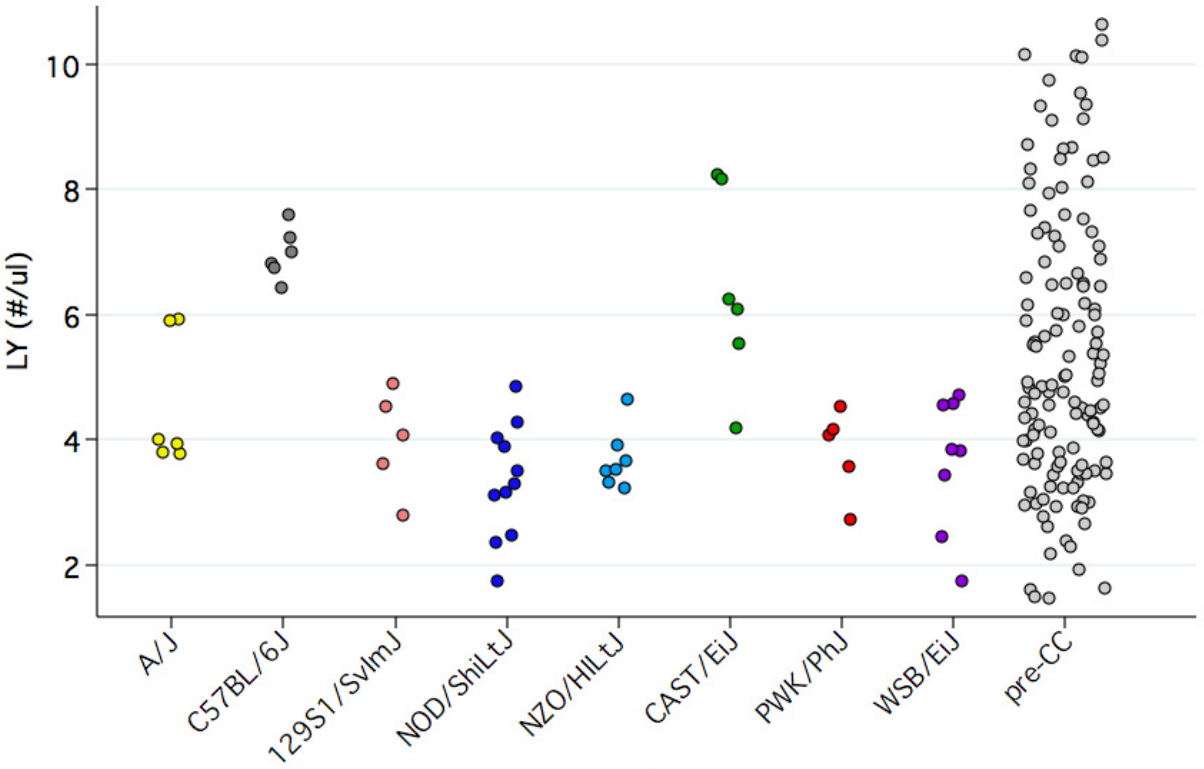
LY (#/ul)
10
8
6
4
2
A/J
C57BL/6J
129S1/SvImJ
NOD/ShiLtJ
NZO/HILtJ
CAST/EiJ
PWK/PhJ
WSB/EiJ
pre-CC

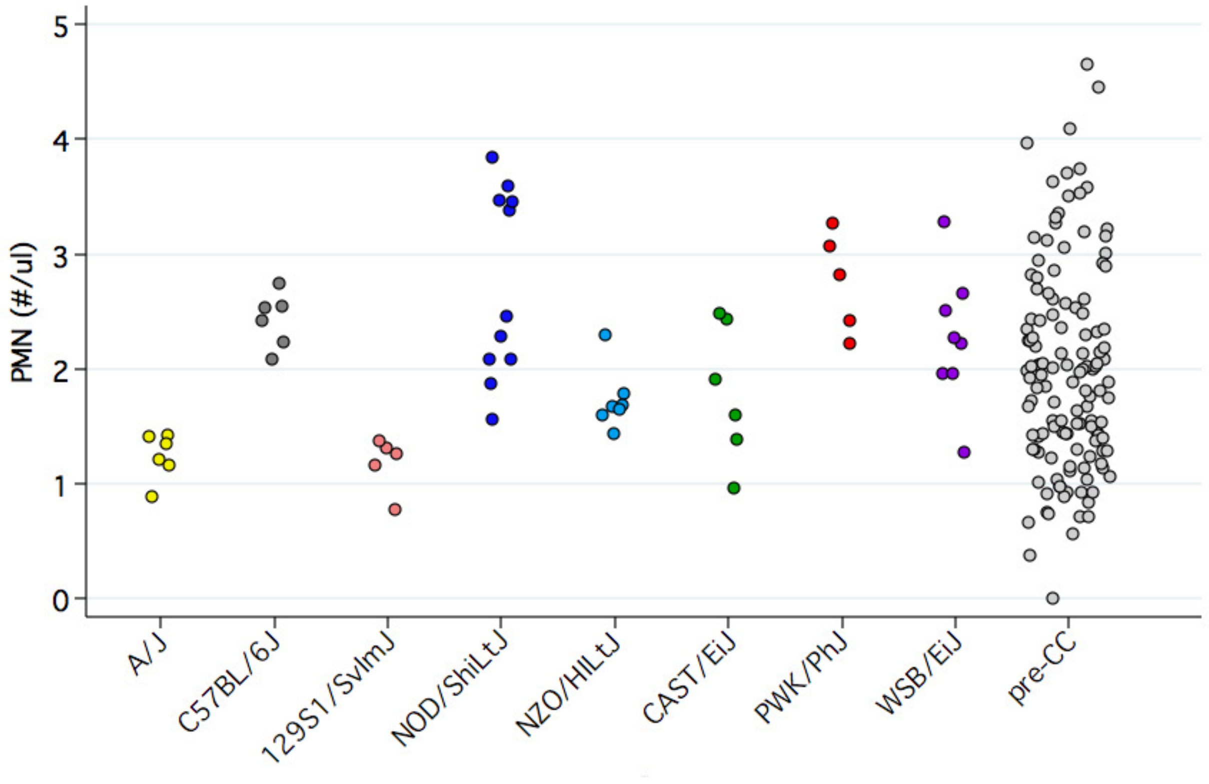

PMN (#/ul)
0
1
2
3
4
5
A/J
C57BL/6J
129S1/SvImJ
NOD/ShiLtJ
NZO/HILtJ
CAST/EiJ
PWK/PhJ
WSB/EiJ
pre-CC

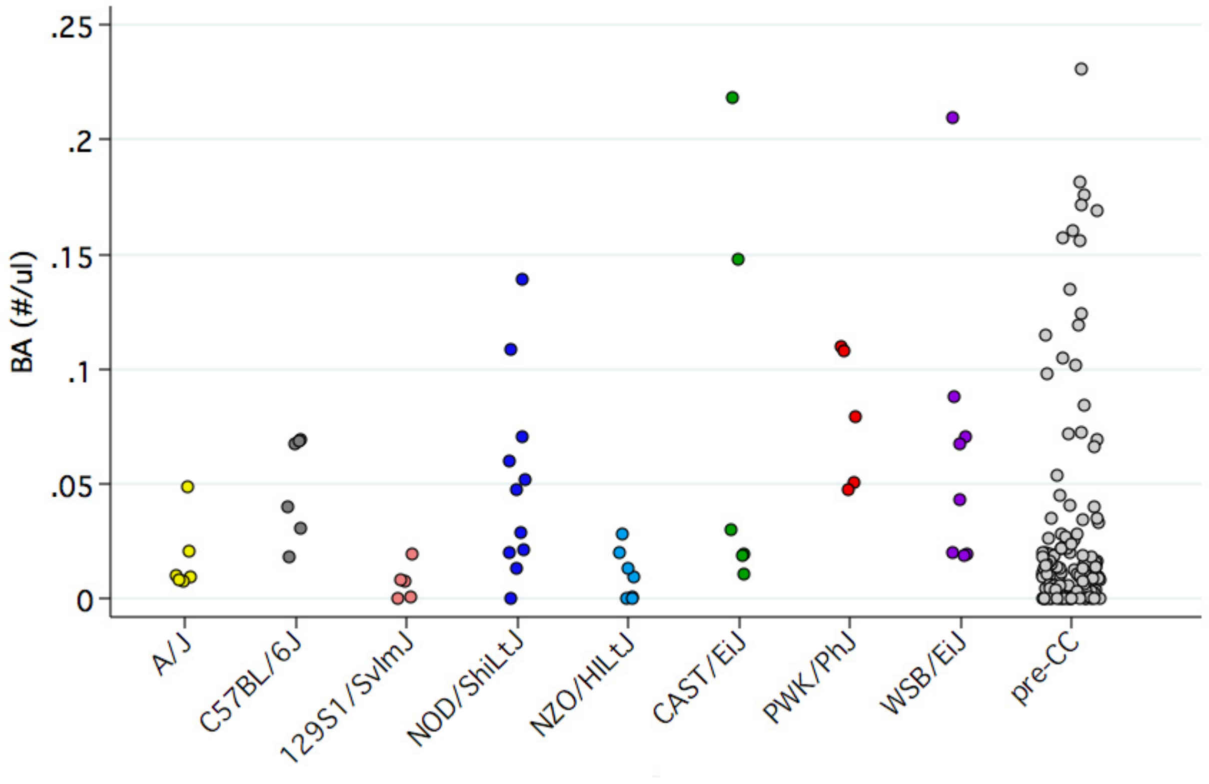

BA (#/ul)
0
.05
.1
.15
.2
.25
A/J
C57BL/6J
129S1/SvImJ
NOD/ShiLtJ
NZO/HILtJ
CAST/EiJ
PWK/PhJ
WSB/EiJ
pre-CC

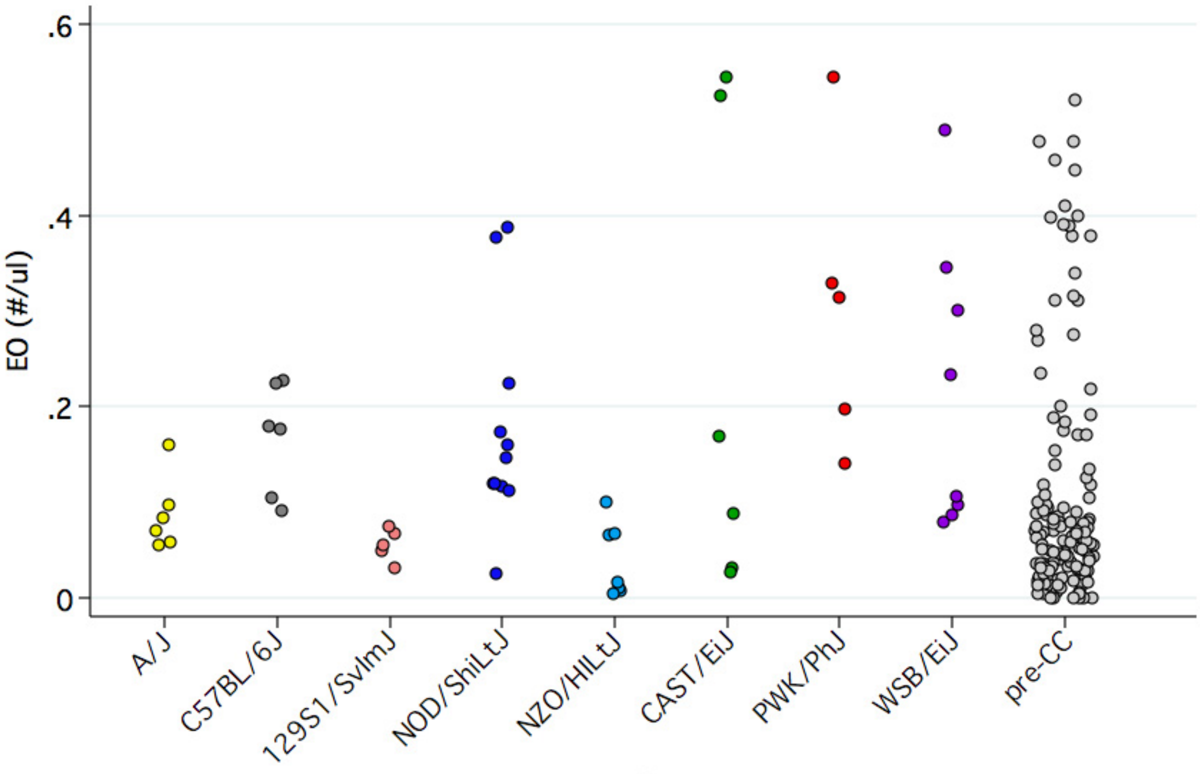

Figure S1 Distributions of hematological traits in pre-CC mice and founder lines for which no QTL were detected.

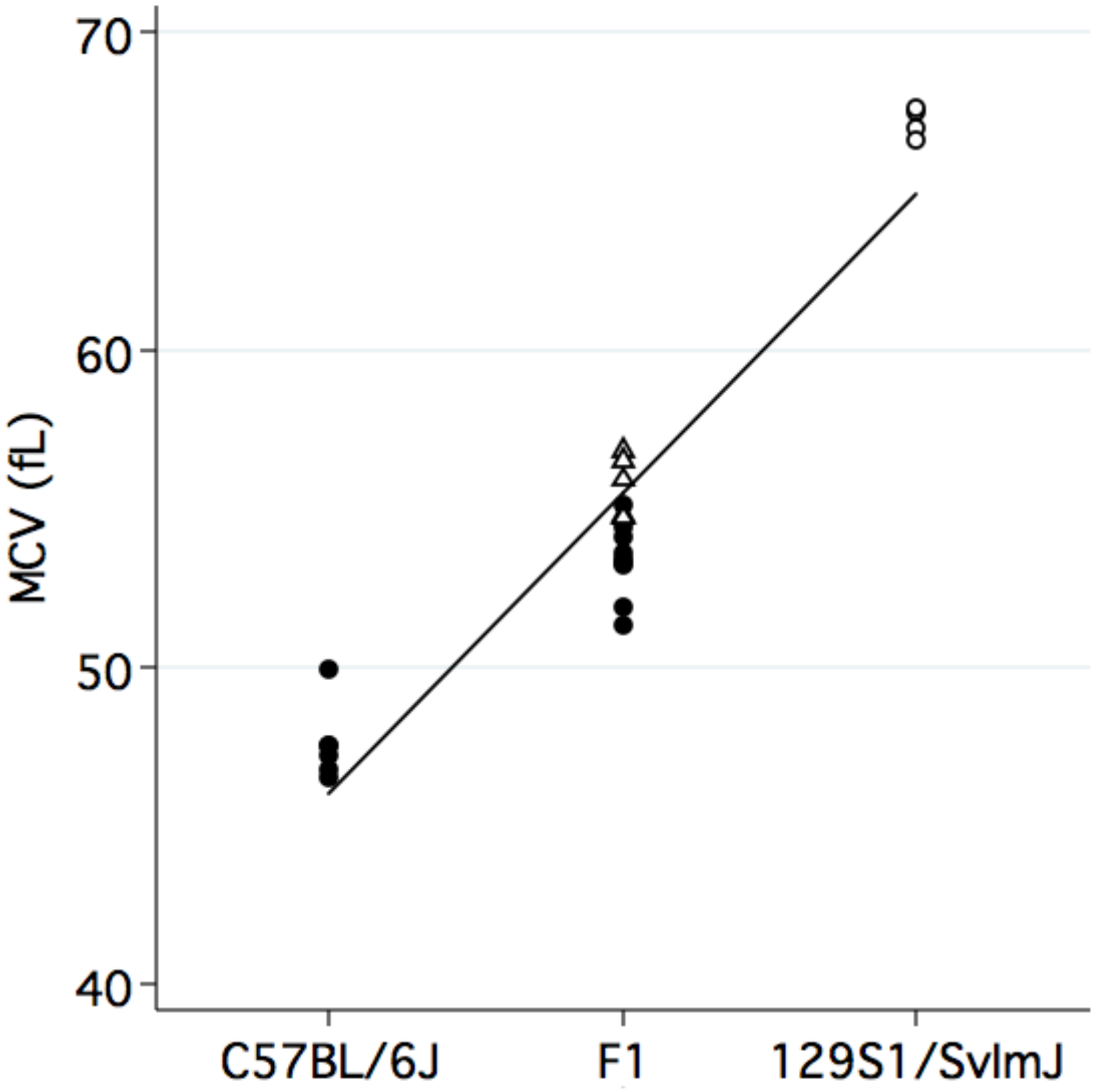

Figure S2 MCV values among C57BL/6J (*Hbb s/s* genotype), 129S1/SvImJ (*Hbb d/d* genotype), and F1 mice (*s/d* genotype). F1 mice from 129S1/SvImJ dams are depicted as white triangles and F1 mice from C57BL/6J dams are depicted as black circles. F1 mice from 129S1/SvImJ dams had larger MCV than F1 mice from the reciprocal cross (55.6 ± 0.4 vs 53.4 ± 0.4 fL, $p = 0.002$).

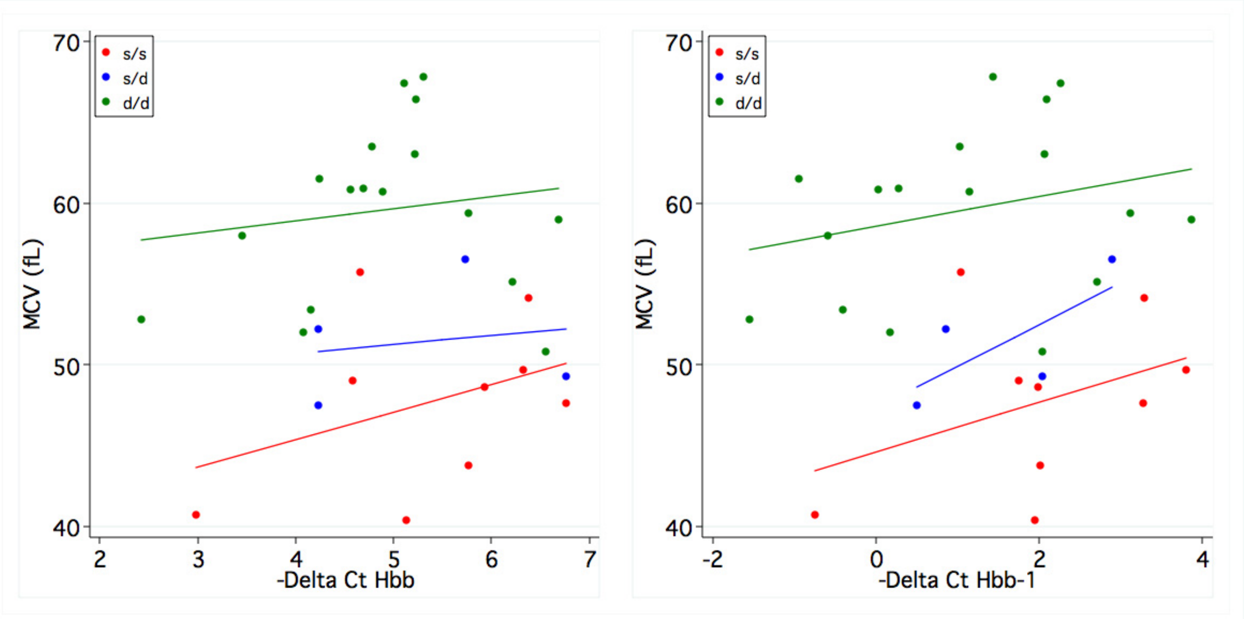

Figure S3 *Hbb* gene expression and MCV in pre-CC mice. Total *Hbb*-β (left) and *Hbb-b1* (right) gene expression were measured by qRT-PCR using spleen RNA from 30 pre-CC mice. Parameters for regression lines are shown in Table S9.

Tables S1 – S3

Tables S1-S3 are available for download at http://www.g3journal.org/lookup/suppl/doi:10.1534/g3.111.001776/-/DC1.

.

Table S4   RBC and WBC Parameters in Collaborative Cross Founder Strain and pre-CC Mice

Strain	RBC (#/uL)	Hb (g/dL)	MCV (fL)	RDW (%)	PLT[1] (#/uL)	MPV (fL)	WBC[1] (#/uL)	LY[1] (#/uL)	NE[1] (#/uL)	MO[1] (#/uL)
**A/J**	9.70 (0.13)	13.83 (0.21)	48.62 (0.22)	19.47 (0.14)	1016.17 (10.64)	5.25 (0.06)	6.34 (0.48)	4.61 (0.43)	1.27 (0.07)	0.35 (0.03)
**C57BL/6J**	10.48 (0.25	15.50 (0.38)	45.97 (0.16)	18.38 (0.16	946.50 (87.17)	5.32 (0.08v	10.74 (0.26)	7.03 (0.16)	2.46 (0.09)	1.03 (0.06v
**129S1/SvImJ**	9.25 (0.74)	13.47 (1.17)	62.42 (0.38)	17.00 (0.25)	449.17 (29.98)	4.25 (0.07)	5.16 (0.64)	3.72 (0.45)	1.11 (0.14)	0.28 (0.05)
**NOD/ShiLtJ**	9.44 (0.27)	15.43 (0.46)	55.90 (0.61)	17.14 (0.14)	896.50 (50.82)	5.18 (0.11)	7.11 (0.59)	3.52 (0.30)	2.86 (0.25)	0.49 (0.04)
**NZO/HILtJ**	10.54 (0.35)	14.93 (0.54)	44.52 (0.30)	20.41 (0.82)	1443.50 (35.41)	5.88 (0.07)	5.90 (0.27)	3.76 (0.20)	1.65 (0.07)	0.48 (0.06)
**CAST/EiJ**	12.18 (0.22)	16.52 (0.33)	46.32 (0.18)	20.05 (0.44)	659.33 (50.81)	4.28 (0.09)	9.04 (0.92)	6.47 (0.64)	1.83 (0.25)	0.44 (0.08)
**PWK/PhJ**	12.93 (0.48	18.54 (0.82	60.84 (0.67	18.56 (0.25)	689.00 (141.28)	5.26 (0.12	8.03 (0.59)	3.87 (0.33)	2.79 (0.20)	0.98 (0.09)
**WSB/EiJ**	12.47 (0.52)	17.15 (0.68)	57.84 (0.30)	16.51 (0.48)	736.13 (30.40)	3.98 (0.03)	7.06 (0.61)	3.67 (0.37)	2.28 (0.21)	0.82 (0.08)
**Pre-CC**	10.12 (0.14)	14.49 (0.18)	54.35 (0.61)	18.15 (0.13)	997.78 (34.80)	4.80 (0.04)	8.00 (0.26)	5.27 (0.19)	2.03 (0.08)	0.56 (0.02)
**F-statistic[2]**	10.89	5.11	260.51	13.37	14.92	58.24	6.46	8.91	11.83	13.55
**p-value**	4.49E-08	2.29E-04	2.29E-35	2.88E-09	4.56E-10	5.11E-21	2.38E-05	6.14E-07	1.42E-08	1.96E-09
**Broad sense heritability[3]**										
r_1	0.62	0.41	0.98	0.67	0.70	0.91	0.48	0.57	0.64	0.68
g^2	0.45	0.25	0.96	0.51	0.54	0.83	0.31	0.40	0.47	0.51

Values are reported as mean (standard error).
[1] data were log-transformed for statistical analysis and mapping.
[2] ANOVA conducted using only founder strains.
[3] r_1, the interclass correlation, and g^2, coefficient of genetic determination, were calculated using founder strains only, as described in (Xing et al., 2009).

Table S5 SNPs in the Hemoglobin β Locus that Distinguish Single vs. Diffuse Strains

**Hbb-b1**	**Amino Acid Position**										
**Strain**	**9**	**13**	**16**	**20**	**58**	**76**	**77**	**80**	**109**	**139**	**Phenotype***
C57BL/6J	A	G	G	A	A	N	H	S	M	A	Single
NOD/ShiLtJ	-	-	-	-	-	-	-	-	-	-	Single
NZO/HiLtJ	-	-	-	-	-	-	-	-	-	-	Single
129S1/SvImJ	S	**C**	A	P	-	-	-	-	-	**T**	Diffuse
A/J	S	**C**	A	P	-	-	-	-	V	**T**	Diffuse
WSB/EiJ	S	**C**	A	S	P	-	-	-	V	**T**	Diffuse
PWK/PhJ	S	**C**	A	P	-	K	N	-	V	**T**	Diffuse
CAST/EiJ	S	**C**	-	-	-	K	N	N	V	**T**	Diffuse

**Hbb-b2**	**Amino Acid Position**										
**Strain**	**9**	**13**	**16**	**20**	**58**	**76**	**77**	**80**	**109**	**139**	**Phenotype***
C57BL/6J	-	-	-	-	-	-	-	-	-	-	Single
NOD/ShiLtJ	-	-	-	-	-	-	-	-	-	-	Single
NZO/HiLtJ	-	-	-	-	-	-	-	-	-	-	Single
129S1/SvImJ	-	**C**	-	S	-	-	-	-	-	**T**	Diffuse
A/J	-	**C**	-	S	-	-	-	-	-	**T**	Diffuse
WSB/EiJ	-	**C**	-	S	-	-	-	-	-	**T**	Diffuse
PWK/PhJ	-	**C**	-	S	-	-	-	-	-	**T**	Diffuse
CAST/EiJ	-	**C**	-	-	-	-	-	-	-	**T**	Diffuse

* Phenotype refers to the band pattern of hemoglobin β protein on isoelectric focusing experiments. Data from literature and databases was compiled to assemble this table.

Table S6 MCV (fL) Among Pre-CC mice and Founder Strains as a Function of *Hbb-b1/b2 Single* versus *Diffuse* Genotypes

Founder Allele	n	Pre-CC	Founder Mice*	p-value
**C57BL/6J (s/s)**	15	46.95	45.97	0.60
**NOD/ShiLtJ (s/s)**	10	47.23	55.90	$\mathbf{2.0x10^{-5}}$
**NZO/HILtJ (s/s)**	9	47.71	44.52	0.28
**A/J (d/d)**	12	58.68	48.62	$8.0\mathbf{x10^{-4}}$
**129S1/SvImJ (d/d)**	7	58.30	62.42	0.15
**CAST/EiJ (d/d)**	9	56.82	46.32	$\mathbf{1.7x10^{-4}}$
**PWK/PhJ (d/d)**	9	61.53	60.84	0.58
**WSB/EiJ (d/d)**	14	60.77	57.84	0.05
**Genotype Averages**				
**s/s**	40	48.15	55.93	
**d/d**	69	59.45	50.46	

* Data for founder mice is from Table S1.

Table S7 Regions of Shared Ancestry Identified Using Mouse Genomes Project SNP Data

QTL	Chr	Start	End	Range	Number of SNPs	Strains with region of shared ancestry	Mismatched SNPs		Shared SNPs		Uniquely Shared SNPs	
							n	%	*n*	%	*n*	%
Mcvq4	7	110959072	111086522	127450	3499	C57BL/6J<br>NOD/HiLtJ<br>NZO/ShiLtJ	27	0.8	3472	99.2	1455	41.6
Mcvq5	14	20453181	22963216	2510035	35931	129S1/SvImJ NOD/HiLtJ	457	1.3	35,474	98.7	619	1.7
Moq1	1	92803891	92970550	166659	3357	A/J<br>WSB/EiJ	76	2.3	3281	97.7	9	0.3

Table S8

Table S8 is available for download as a text file at http://www.g3journal.org/lookup/suppl/doi:10.1534/g3.111.001776/-/DC1.

Table S9 Regression Models of MCV as a Function of Hbb s/d Genotype and Hbb Gene Expression*

1. Hbb gene expression		
Term	Beta	p-value
Hbb expression	-0.02	0.99
Intercept	55.02	<0.001

2. Hbb gene expression and s/d genotype		
Predictor	Beta	p-value
Hbb expression	1.00	0.26
Genotype	6.26	<0.001
Intercept	41.88	<0.001

3. Hbb gene expression, s/d genotype, and genotype*expression		
Predictor	Beta	p-value
Hbb expression	1.62	0.28
Genotype	8.89	0.10
Genotype*Expression	-0.51	0.61
Intercept	38.56	<0.001

4. Hbb-b1 gene expression		
Term	Beta	p-value
Hbb-b1 expression	0.00	1.00
Intercept	54.91	<0.001

5. Hbb-b1 and s/d genotype		
	Beta	p-value
Gene Expression	1.20	0.08
Genotype	6.57	<0.001
Intercept	44.87	<0.001

6. Hbb-b1 gene expression, s/d genotype, and genotype*expression		
Hbb-b1 expression	1.79	0.16
Genotype	7.31	<0.001
Genotype*Expression	-0.43	0.58
Intercept	43.69	<0.001

* Note that *Hbb* gene expression (total or Hbb-b1) is expressed as the ΔCt (relative to *Rps29*) –ΔCt as in Figure S3.
