# Supplementary material for: Genetic Analysis of Hematological Parameters in Incipient Lines of the Collaborative Cross
Source: G3 (Bethesda). 2012 Feb 1;2(2):157–65. doi: 10.1534/g3.111.001776 (PMC3284323; doi:10.1534/g3.111.001776)
Supplement: Supporting Information [file supp_2.2.157_FigureS1.pdf]

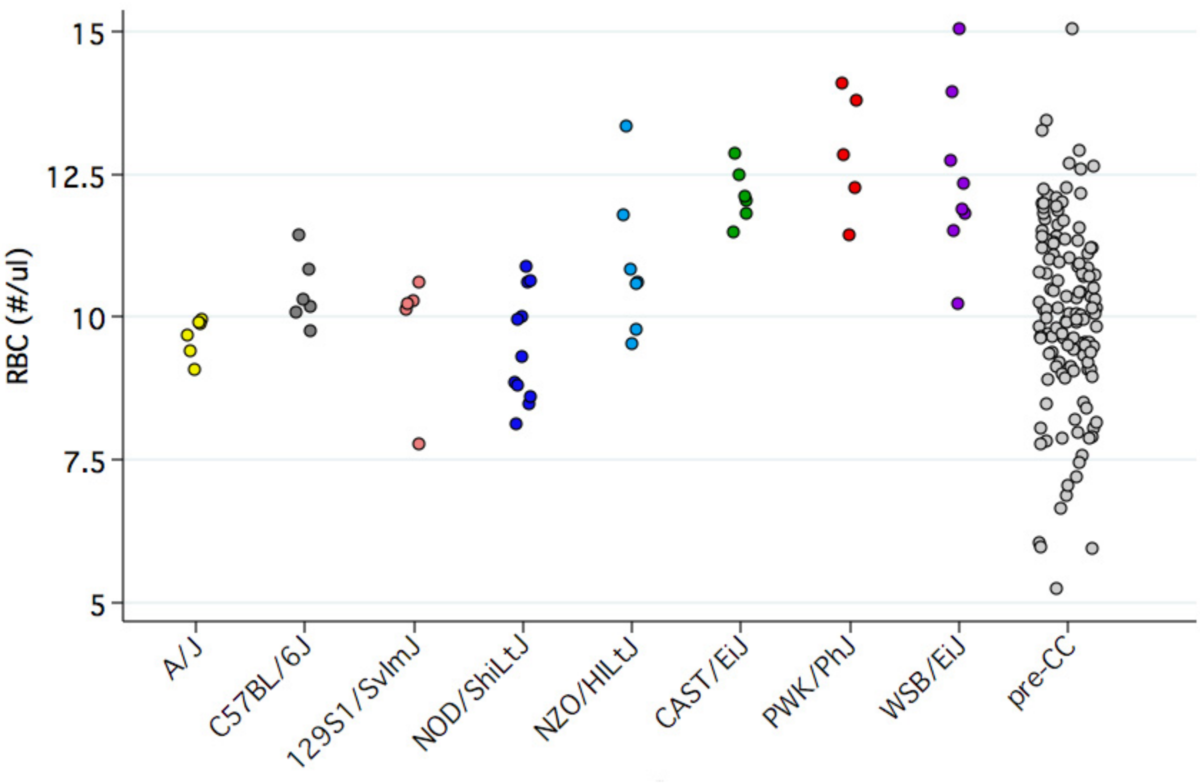

RBC (#/ul)
15
12.5
10
7.5
5
A/J
C57BL/6J
129S1/SvImJ
NOD/ShiLtJ
NZO/HILtJ
CAST/EiJ
PWK/PhJ
WSB/EiJ
pre-CC

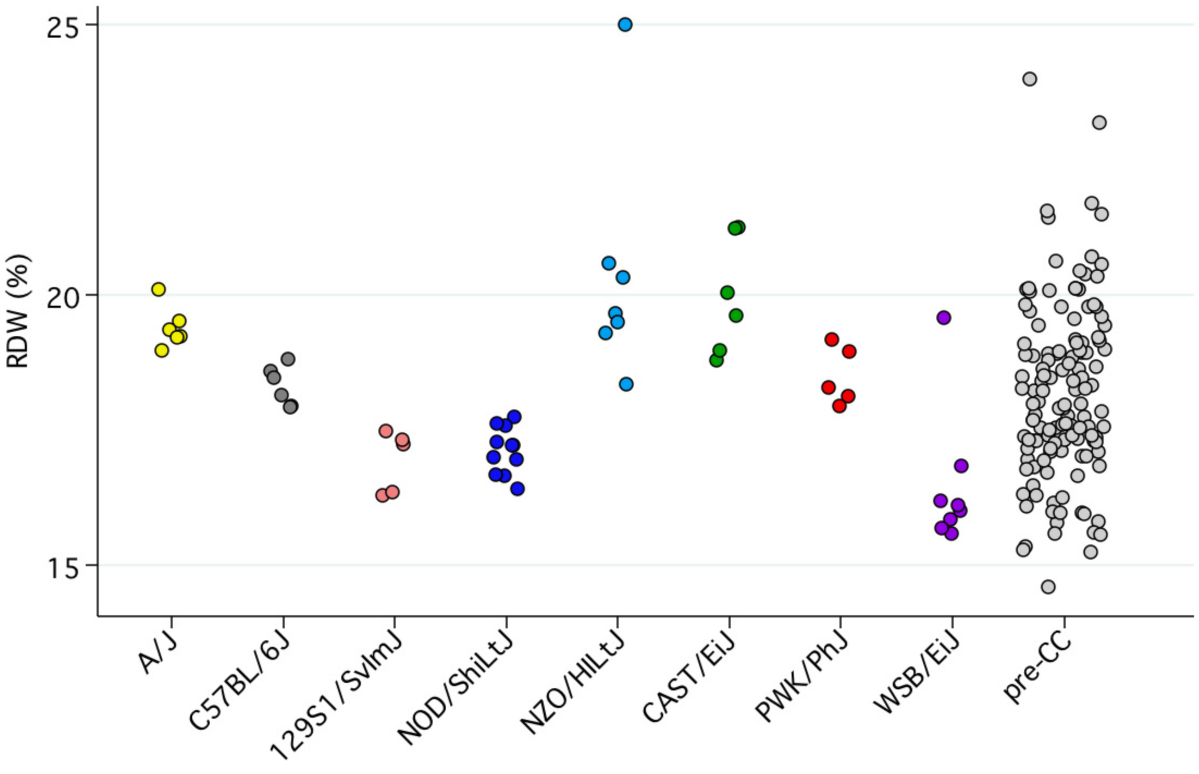

RDW (%)
25
20
15
A/J
C57BL/6J
129S1/SvImJ
NOD/ShiLtJ
NZO/HILtJ
CAST/EiJ
PWK/PhJ
WSB/EiJ
pre-CC

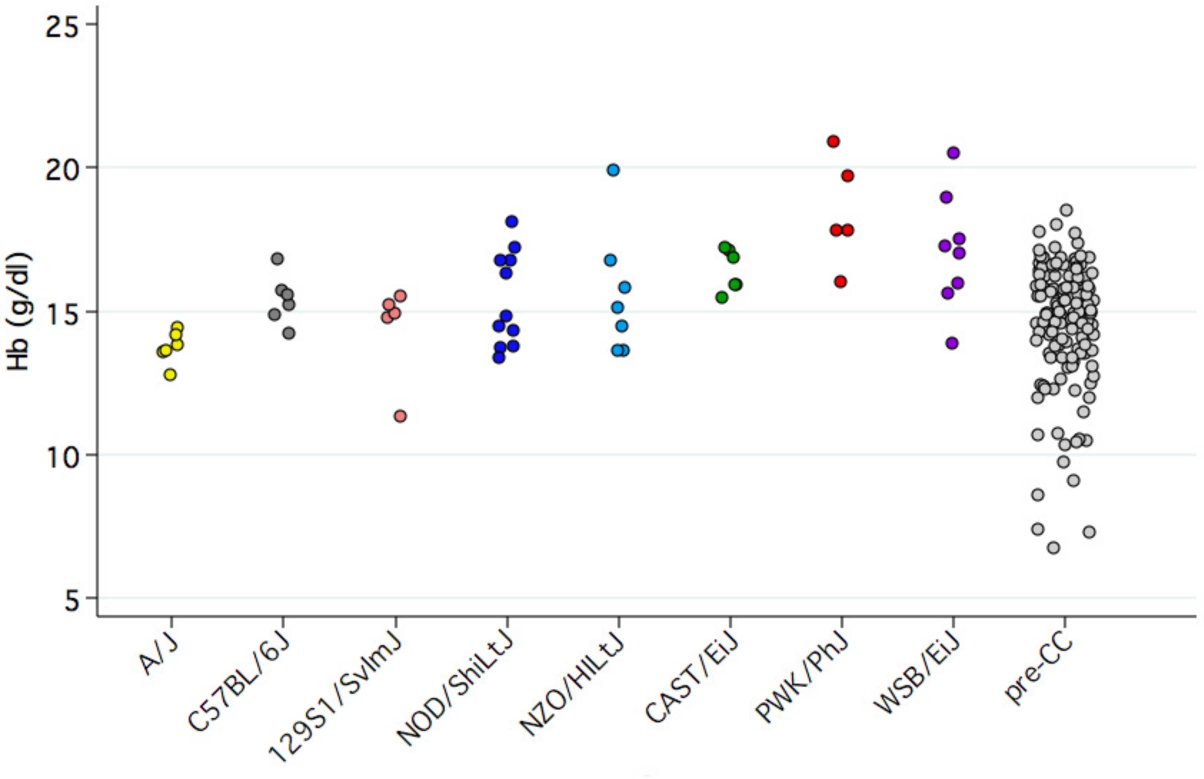
25
20
15
10
5
Hb (g/dl)
A/J
C57BL/6J
129S1/SvImJ
NOD/ShiLtJ
NZO/HILtJ
CAST/EiJ
PWK/PhJ
WSB/EiJ
pre-CC

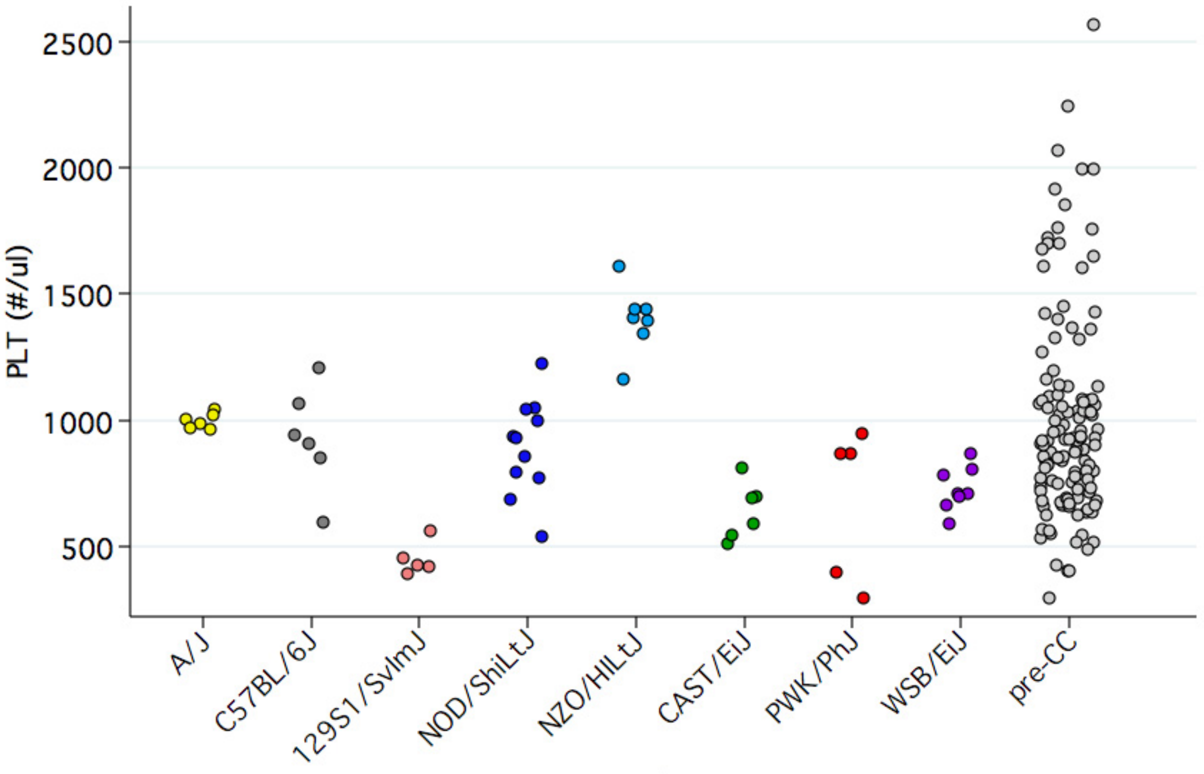
2500
2000
1500
1000
500
PLT (#/ul)
A/J
C57BL/6J
129S1/SvImJ
NOD/ShiLtJ
NZO/HILtJ
CAST/EiJ
PWK/PhJ
WSB/EiJ
pre-CC

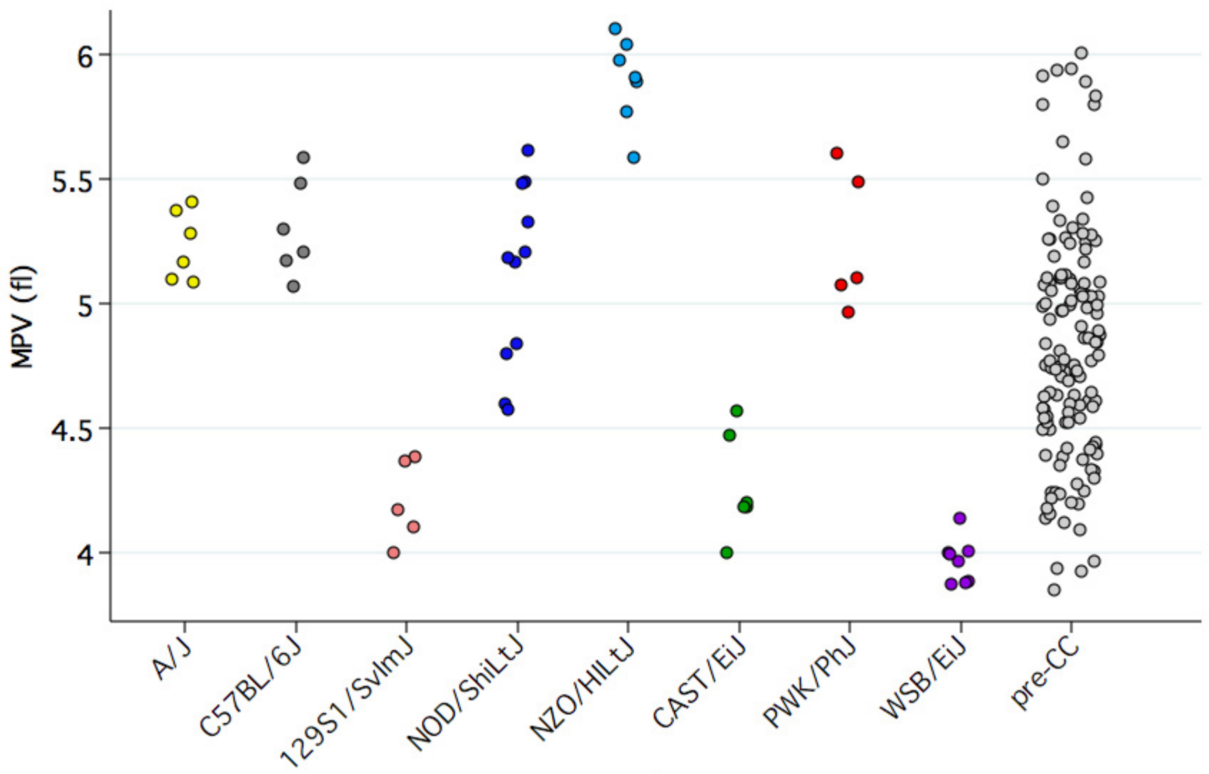
MPV (fl)
6
5.5
5
4.5
4
A/J
C57BL/6J
129S1/SvImJ
NOD/ShiLtJ
NZO/HlLtJ
CAST/EiJ
PWK/PhJ
WSB/EiJ
pre-CC

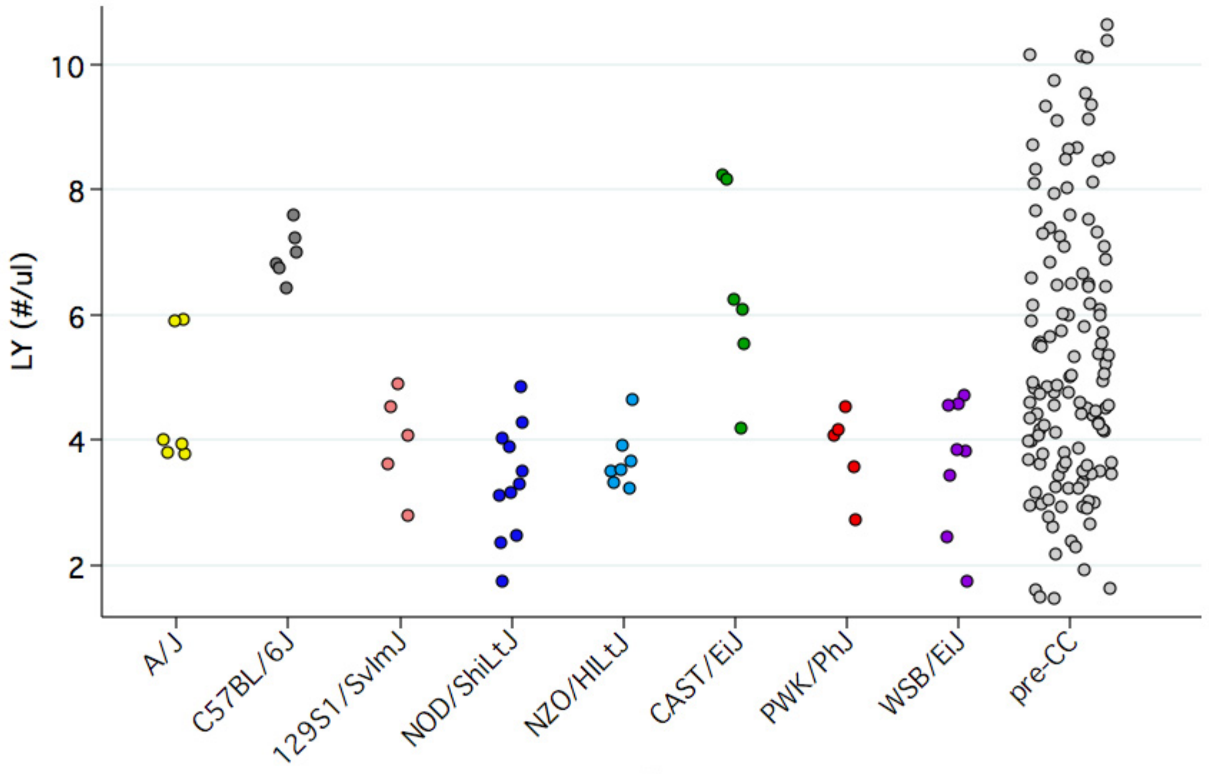
LY (#/ul)
10
8
6
4
2
A/J
C57BL/6J
129S1/SvImJ
NOD/ShiLtJ
NZO/HlLtJ
CAST/EiJ
PWK/PhJ
WSB/EiJ
pre-CC

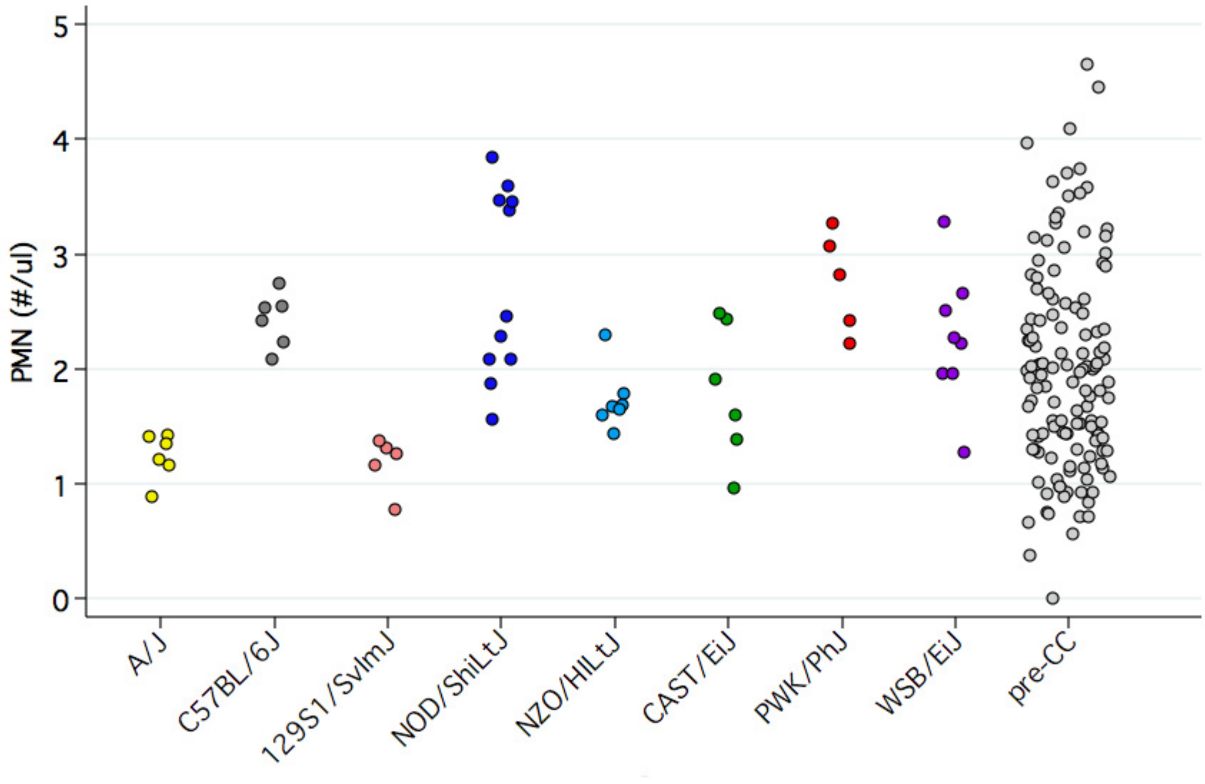
PMN (#/ul)
0
1
2
3
4
5
A/J
C57BL/6J
129S1/SvImJ
NOD/ShiLtJ
NZO/HILtJ
CAST/EiJ
PWK/PhJ
WSB/EiJ
pre-CC

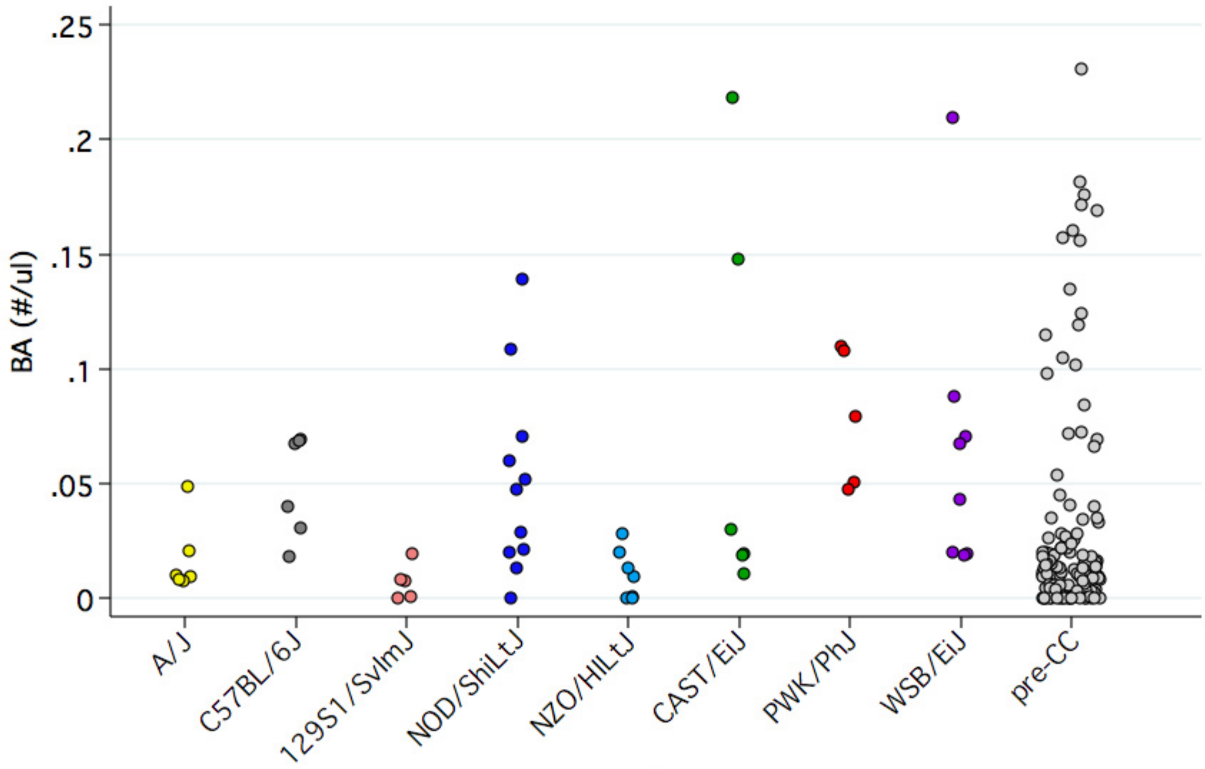
BA (#/ul)
0
.05
.1
.15
.2
.25
A/J
C57BL/6J
129S1/SvImJ
NOD/ShiLtJ
NZO/HILtJ
CAST/EiJ
PWK/PhJ
WSB/EiJ
pre-CC

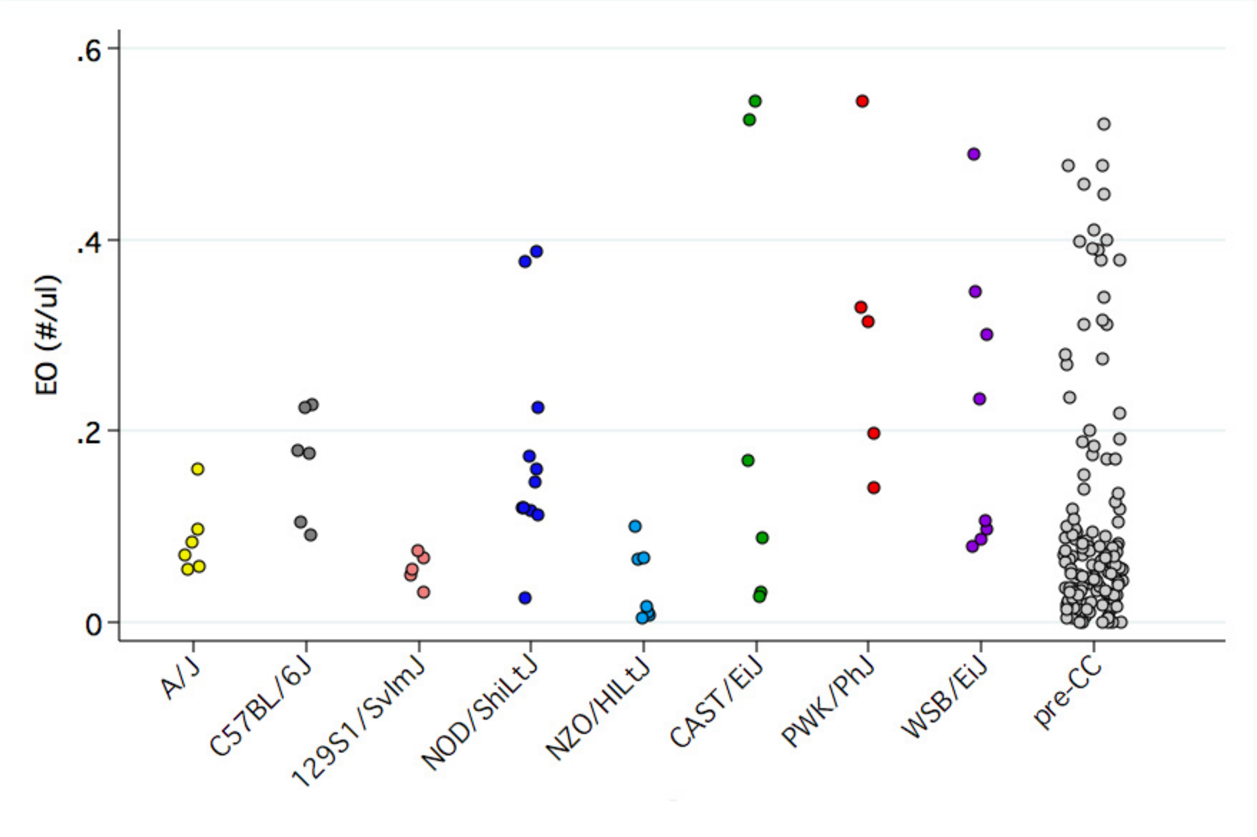

Figure S1 Distributions of hematological traits in pre-CC mice and founder lines for which no QTL were detected.
