# Supplementary material for: Genetic Analysis of Hematological Parameters in Incipient Lines of the Collaborative Cross
Source: G3 (Bethesda). 2012 Feb 1;2(2):157–65. doi: 10.1534/g3.111.001776 (PMC3284323; doi:10.1534/g3.111.001776)
Supplement: Supporting Information [file supp_2.2.157_FigureS2.pdf]

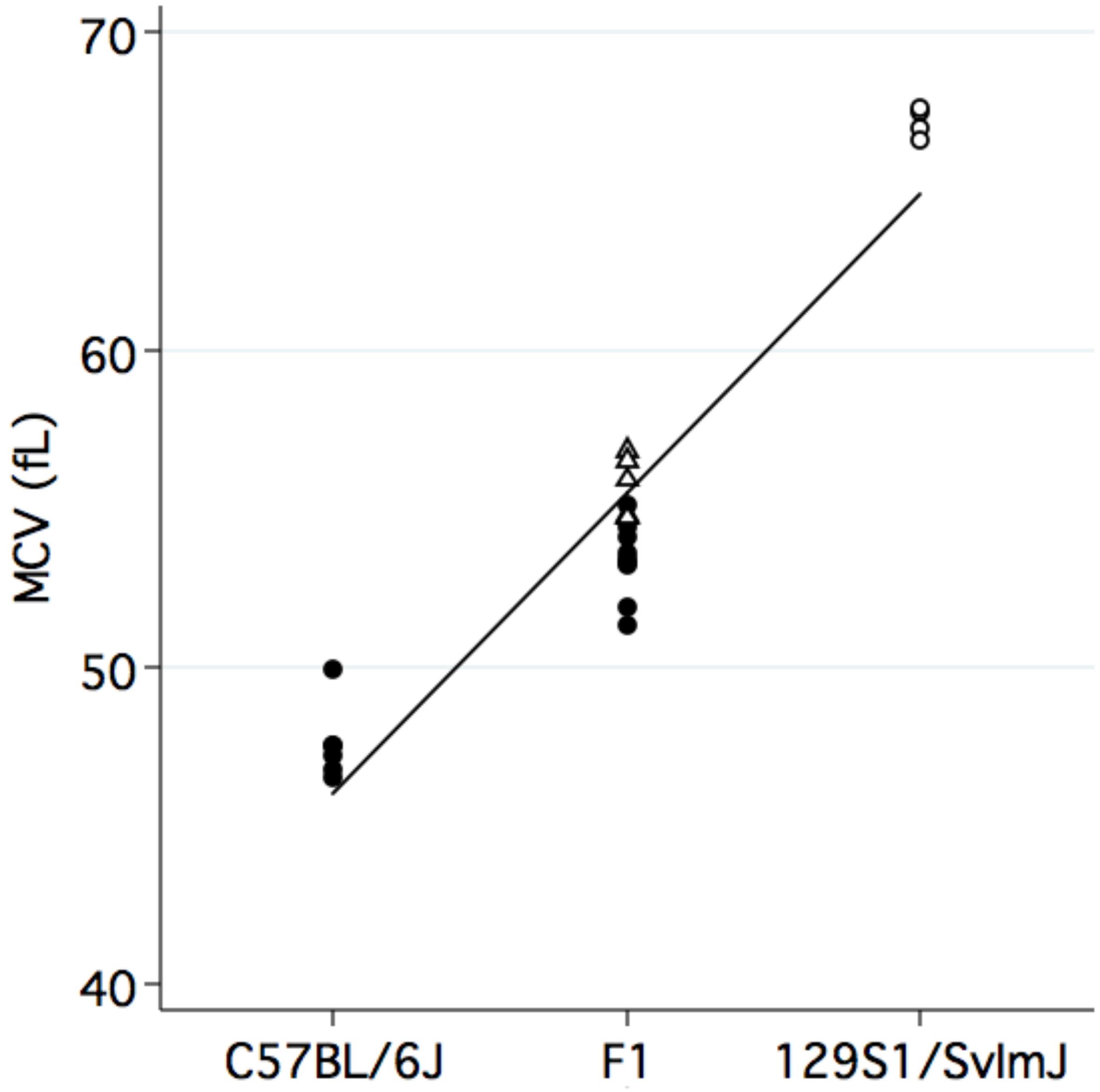

Figure S2 MCV values among C57BL/6J (*Hbb s/s* genotype), 129S1/SvImJ (*Hbb d/d* genotype), and F1 mice (*s/d* genotype). F1 mice from 129S1/SvImJ dams are depicted as white triangles and F1 mice from C57BL/6J dams are depicted as black circles. F1 mice from 129S1/SvImJ dams had larger MCV than F1 mice from the reciprocal cross (55.6 ± 0.4 vs 53.4 ± 0.4 fL, p = 0.002).
