# Supplementary material for: Genetic Analysis of Hematological Parameters in Incipient Lines of the Collaborative Cross
Source: G3 (Bethesda). 2012 Feb 1;2(2):157–65. doi: 10.1534/g3.111.001776 (PMC3284323; doi:10.1534/g3.111.001776)
Supplement: Supporting Information [file supp_2.2.157_FigureS3.pdf]

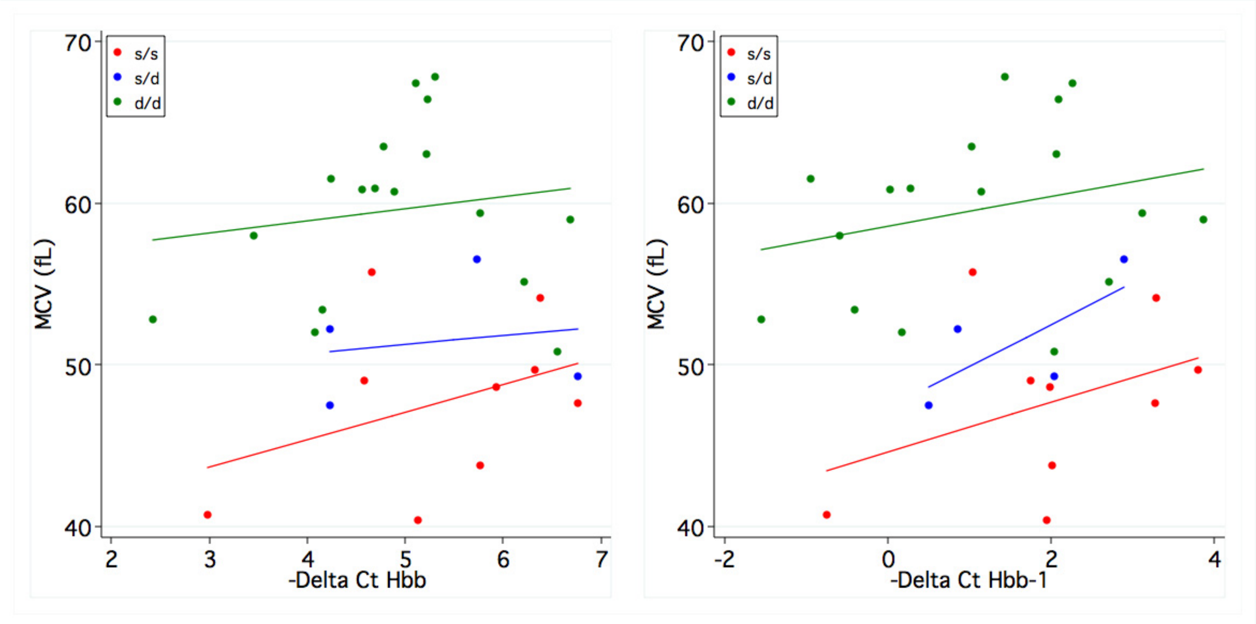

Figure S3 *Hbb* gene expression and MCV in pre-CC mice. Total *Hbb*-β (left) and *Hbb-b1* (right) gene expression were measured by qRT-PCR using spleen RNA from 30 pre-CC mice. Parameters for regression lines are shown in Table S9.
