# Supplementary material for: Genetic Analysis of Hematological Parameters in Incipient Lines of the Collaborative Cross
Source: G3 (Bethesda). 2012 Feb 1;2(2):157–65. doi: 10.1534/g3.111.001776 (PMC3284323; doi:10.1534/g3.111.001776)
Supplement: Supporting Information [file supp_2.2.157_TableS4.pdf]

Table S4 RBC and WBC Parameters in Collaborative Cross Founder Strain and pre-CC Mice

**Strain**	RBC (#/uL)	Hb (g/dL)	MCV (fL)	RDW (%)	PLT[1] (#/uL)	MPV (fL)	WBC[1] (#/uL)	LY[1] (#/uL)	NE[1] (#/uL)	MO[1] (#/uL)
**A/J**	9.70 (0.13)	13.83 (0.21)	48.62 (0.22)	19.47 (0.14)	1016.17 (10.64)	5.25 (0.06)	6.34 (0.48)	4.61 (0.43)	1.27 (0.07)	0.35 (0.03)
**C57BL/6J**	10.48 (0.25	15.50 (0.38)	45.97 (0.16)	18.38 (0.16	946.50 (87.17)	5.32 (0.08v	10.74 (0.26)	7.03 (0.16)	2.46 (0.09)	1.03 (0.06v
**129S1/SvImJ**	9.25 (0.74)	13.47 (1.17)	62.42 (0.38)	17.00 (0.25)	449.17 (29.98)	4.25 (0.07)	5.16 (0.64)	3.72 (0.45)	1.11 (0.14)	0.28 (0.05)
**NOD/ShiLtJ**	9.44 (0.27)	15.43 (0.46)	55.90 (0.61)	17.14 (0.14)	896.50 (50.82)	5.18 (0.11)	7.11 (0.59)	3.52 (0.30)	2.86 (0.25)	0.49 (0.04)
**NZO/HILtJ**	10.54 (0.35)	14.93 (0.54)	44.52 (0.30)	20.41 (0.82)	1443.50 (35.41)	5.88 (0.07)	5.90 (0.27)	3.76 (0.20)	1.65 (0.07)	0.48 (0.06)
**CAST/EiJ**	12.18 (0.22)	16.52 (0.33)	46.32 (0.18)	20.05 (0.44)	659.33 (50.81)	4.28 (0.09)	9.04 (0.92)	6.47 (0.64)	1.83 (0.25)	0.44 (0.08)
**PWK/PhJ**	12.93 (0.48	18.54 (0.82	60.84 (0.67	18.56 (0.25)	689.00 (141.28)	5.26 (0.12	8.03 (0.59)	3.87 (0.33)	2.79 (0.20)	0.98 (0.09)
**WSB/EiJ**	12.47 (0.52)	17.15 (0.68)	57.84 (0.30)	16.51 (0.48)	736.13 (30.40)	3.98 (0.03)	7.06 (0.61)	3.67 (0.37)	2.28 (0.21)	0.82 (0.08)
**Pre-CC**	10.12 (0.14)	14.49 (0.18)	54.35 (0.61)	18.15 (0.13)	997.78 (34.80)	4.80 (0.04)	8.00 (0.26)	5.27 (0.19)	2.03 (0.08)	0.56 (0.02)
**F-statistic**[2]	10.89	5.11	260.51	13.37	14.92	58.24	6.46	8.91	11.83	13.55
**p-value**	4.49E-08	2.29E-04	2.29E-35	2.88E-09	4.56E-10	5.11E-21	2.38E-05	6.14E-07	1.42E-08	1.96E-09
**Broad sense heritability**[3]										
**r_1**	0.62	0.41	0.98	0.67	0.70	0.91	0.48	0.57	0.64	0.68
**g^2**	0.45	0.25	0.96	0.51	0.54	0.83	0.31	0.40	0.47	0.51

Values are reported as mean (standard error).
[1] data were log-transformed for statistical analysis and mapping.
[2] ANOVA conducted using only founder strains.
[3] r_1, the interclass correlation, and g^2, coefficient of genetic determination, were calculated using founder strains only, as described in (Xing et al., 2009).
