# Supplementary material for: Genetic Analysis of Hematological Parameters in Incipient Lines of the Collaborative Cross
Source: G3 (Bethesda). 2012 Feb 1;2(2):157–65. doi: 10.1534/g3.111.001776 (PMC3284323; doi:10.1534/g3.111.001776)
Supplement: Supporting Information [file supp_2.2.157_TableS5.pdf]

Table S5 SNPs in the Hemoglobin β Locus that Distinguish Single vs. Diffuse Strains

**Hbb-b1**	**Amino Acid Position**										
**Strain**	**9**	**13**	**16**	**20**	**58**	**76**	**77**	**80**	**109**	**139**	**Phenotype***
C57BL/6J	A	G	G	A	A	N	H	S	M	A	Single
NOD/ShiLtJ	-	**-**	-	-	-	-	-	-	-	**-**	Single
NZO/HiLtJ	-	**-**	-	-	-	-	-	-	-	**-**	Single
129S1/SvImJ	S	**C**	A	P	-	-	-	-	-	**T**	Diffuse
A/J	S	**C**	A	P	-	-	-	-	V	**T**	Diffuse
WSB/EiJ	S	**C**	A	S	P	-	-	-	V	**T**	Diffuse
PWK/PhJ	S	**C**	A	P	-	K	N	-	V	**T**	Diffuse
CAST/EiJ	S	**C**	-	-	-	K	N	N	V	**T**	Diffuse

**Hbb-b2**	**Amino Acid Position**										
**Strain**	**9**	**13**	**16**	**20**	**58**	**76**	**77**	**80**	**109**	**139**	**Phenotype***
C57BL/6J	-	**-**	-	-	-	-	-	-	-	**-**	Single
NOD/ShiLtJ	-	**-**	-	-	-	-	-	-	-	**-**	Single
NZO/HiLtJ	-	**-**	-	-	-	-	-	-	-	**-**	Single
129S1/SvImJ	-	**C**	-	S	-	-	-	-	-	**T**	Diffuse
A/J	-	**C**	-	S	-	-	-	-	-	**T**	Diffuse
WSB/EiJ	-	**C**	-	S	-	-	-	-	-	**T**	Diffuse
PWK/PhJ	-	**C**	-	S	-	-	-	-	-	**T**	Diffuse
CAST/EiJ	-	**C**	-	-	-	-	-	-	-	**T**	Diffuse

* Phenotype refers to the band pattern of hemoglobin β protein on isoelectric focusing experiments. Data from literature and databases was compiled to assemble this table.
