# Supplementary material for: Genetic Analysis of Hematological Parameters in Incipient Lines of the Collaborative Cross
Source: G3 (Bethesda). 2012 Feb 1;2(2):157–65. doi: 10.1534/g3.111.001776 (PMC3284323; doi:10.1534/g3.111.001776)
Supplement: Supporting Information [file supp_2.2.157_TableS6.pdf]

Table S6 MCV (fL) Among Pre-CC mice and Founder Strains as a Function of *Hbb-b1/b2 Single* versus *Diffuse* Genotypes

**Founder Allele**	**n**	**Pre-CC**	**Founder Mice***	**p-value**
**C57BL/6J (s/s)**	15	46.95	45.97	0.60
**NOD/ShiLtJ (s/s)**	10	47.23	55.90	**$2.0x10^{-5}$**
**NZO/HILtJ (s/s)**	9	47.71	44.52	0.28
**A/J (d/d)**	12	58.68	48.62	$8.0\mathbf{x10^{-4}}$
**129S1/SvImJ (d/d)**	7	58.30	62.42	0.15
**CAST/EiJ (d/d)**	9	56.82	46.32	**$1.7x10^{-4}$**
**PWK/PhJ (d/d)**	9	61.53	60.84	0.58
**WSB/EiJ (d/d)**	14	60.77	57.84	0.05
**Genotype Averages**				
**s/s**	40	48.15	55.93	
**d/d**	69	59.45	50.46	

* Data for founder mice is from Table S1.
