# Supplementary material for: Genetic Analysis of Hematological Parameters in Incipient Lines of the Collaborative Cross
Source: G3 (Bethesda). 2012 Feb 1;2(2):157–65. doi: 10.1534/g3.111.001776 (PMC3284323; doi:10.1534/g3.111.001776)
Supplement: Supporting Information [file supp_2.2.157_TableS7.pdf]

Table S7 Regions of Shared Ancestry Identified Using Mouse Genomes Project SNP Data

QTL	Chr	Start	End	Range	Number of SNPs	Strains with region of shared ancestry	Mismatched SNPs		Shared SNPs		Uniquely Shared SNPs	
							n	%	*n*	%	*n*	%
Mcvq4	7	110959072	111086522	127450	3499	C57BL/6J<br>NOD/HiLtJ<br>NZO/ShiLtJ	27	0.8	3472	99.2	1455	41.6
Mcvq5	14	20453181	22963216	2510035	35931	129S1/SvImJ NOD/HiLtJ	457	1.3	35,474	98.7	619	1.7
Moq1	1	92803891	92970550	166659	3357	A/J<br>WSB/EiJ	76	2.3	3281	97.7	9	0.3
