# Supplementary material for: Genetic Analysis of Hematological Parameters in Incipient Lines of the Collaborative Cross
Source: G3 (Bethesda). 2012 Feb 1;2(2):157–65. doi: 10.1534/g3.111.001776 (PMC3284323; doi:10.1534/g3.111.001776)
Supplement: Supporting Information [file supp_2.2.157_TableS9.pdf]

Table S9 Regression Models of MCV as a Function of Hbb s/d Genotype and Hbb Gene Expression*

1. Hbb gene expression		
Term	Beta	p-value
Hbb expression	-0.02	0.99
Intercept	55.02	<0.001

2. Hbb gene expression and s/d genotype		
Predictor	Beta	p-value
Hbb expression	1.00	0.26
Genotype	6.26	<0.001
Intercept	41.88	<0.001

3. Hbb gene expression, s/d genotype, and genotype*expression		
Predictor	Beta	p-value
Hbb expression	1.62	0.28
Genotype	8.89	0.10
Genotype*Expression	-0.51	0.61
Intercept	38.56	<0.001

4. Hbb-b1 gene expression		
Term	Beta	p-value
Hbb-b1 expression	0.00	1.00
Intercept	54.91	<0.001

5. Hbb-b1 and s/d genotype		
	Beta	p-value
Gene Expression	1.20	0.08
Genotype	6.57	<0.001
Intercept	44.87	<0.001

6. Hbb-b1 gene expression, s/d genotype, and genotype*expression		
Hbb-b1 expression	1.79	0.16
Genotype	7.31	<0.001
Genotype*Expression	-0.43	0.58
Intercept	43.69	<0.001

* Note that *Hbb* gene expression (total or Hbb-b1) is expressed as the ΔCt (relative to *Rps29*) –ΔCt as in Figure S3.
